# Analysis of CA Content and CPSF6 Dependence of Early HIV-1 Replication Complexes in SupT1-R5 Cells

**DOI:** 10.1128/mBio.02501-19

**Published:** 2019-11-05

**Authors:** Vojtech Zila, Thorsten G. Müller, Vibor Laketa, Barbara Müller, Hans-Georg Kräusslich

**Affiliations:** a Department of Infectious Diseases, Virology, University of Heidelberg, Heidelberg, Germany; b German Center for Infection Research, Heidelberg, Germany; University of Washington

**Keywords:** human immunodeficiency virus, reverse transcription complex, preintegration complex, postentry, capsid, T cells, CPSF6, nuclear pore complex, nuclear import

## Abstract

HIV-1 infects host cells by fusion at the plasma membrane, leading to cytoplasmic entry of the viral capsid encasing the genome and replication machinery. The capsid eventually needs to disassemble, but time and location of uncoating are not fully characterized and may vary depending on the host cell. To study the fate of the capsid by fluorescence and superresolution (STED) microscopy, we established an experimental system that allows discrimination of subviral structures in the cytosol from intact virions at the plasma membrane or in endosomes without genetic modification of the virus. Quantitative microscopy of infected SupT1-R5 cells revealed that the CA signal on cytosolic HIV-1 complexes corresponded to ∼50% of that found in virions at the cell surface, in agreement with dissociation of nonassembled CA molecules from entering capsids after membrane fusion. The relative amount of CA in postfusion complexes remained stable until they reached the nuclear pore complex, while subviral structures in the nucleus of infected cells lacked detectable CA. An HIV-1 variant defective in binding of the host protein cleavage and polyadenylation specificity factor 6 (CPSF6) exhibited accumulation of CA-positive subviral complexes close to the nuclear envelope without loss of infectivity; STED microscopy revealed direct association of these complexes with nuclear pores. These results support previous observations indicating capsid uncoating at the nuclear pore in infected T-cell lines. They suggest that largely intact HIV-1 capsids dock at the nuclear pore in infected SupT1-R5 cells, with CPSF6 being a facilitator of nucleoplasmic entry in this cell type, as has been observed for infected macrophages.

## Abstract (precis)

The HIV-1 capsid performs essential functions during early viral replication and is an interesting target for novel antivirals. Thus, understanding molecular and structural details of capsid function will be important for elucidating early HIV-1 (and retroviral in general) replication in relevant target cells and may also aid antiviral development. Here, we show that HIV-1 capsids stay largely intact during transport to the nucleus of infected T cells but appear to uncoat upon entry into the nucleoplasm. These results support the hypothesis that capsids protect the HIV-1 genome from cytoplasmic defense mechanisms and target the genome toward the nucleus. A protective role of the capsid could be a paradigm that also applies to other viruses. Our findings raise the question of how reverse transcription of the HIV-1 genome is accomplished in the context of the capsid structure and whether the process is completed before the capsid is uncoated at the nuclear pore.

## INTRODUCTION

Human immunodeficiency virus type 1 (HIV-1) is an enveloped retrovirus that enters target cells by fusion of viral and cellular membranes, mediated by specific interactions of the viral envelope (Env) glycoproteins with the cellular receptor CD4 (1) and one of two coreceptors (CXCR4 or CCR5) (2, 3). Membrane fusion leads to release of the viral capsid into the cytosol of the target cell. The mature HIV-1 capsid is a cone-shaped structure with fullerene geometry, consisting of ∼1,200 copies of the CA (capsid) protein (reviewed in reference 4); this represents approximately one-half of the total number of CA molecules inside the virion (5–7). The capsid encases two copies of genomic RNA in a ribonucleoprotein complex with the NC (nucleocapsid) protein, the replication proteins reverse transcriptase (RT) and integrase (IN), as well as the accessory protein Vpr. Once inside the cell, the HIV-1 genome is reverse transcribed into double-stranded cDNA, transported into the nucleus through the nuclear pore complex (NPC), and integrated into host cell DNA. Reverse transcription, intracellular transport, and integration occur via incompletely defined nucleoprotein complexes, termed reverse transcription complex (RTC) and preintegration complex (PIC), respectively (for reviews, see references 8 and 9).

Several lines of evidence have accumulated in recent years, indicating that CA and/or the structure of the viral capsid are important during the critical steps of early HIV-1 postentry replication, including reverse transcription, trafficking toward the nucleus, evasion from the cell-autonomous innate immune response, and nuclear entry (reviewed in references 10–13). While the capsid was initially assumed to dissociate rapidly upon membrane fusion, it is now generally accepted that this is not the case. However, CA has to, at least partially, dissociate at some point during early replication to allow the release of the PIC for genome integration. Furthermore, the size of the capsid (14, 15) extends beyond the exclusion limit of the nuclear pore (16, 17), suggesting that the capsid uncoats or becomes remodeled before nuclear entry, or that the nuclear pore can undergo remodeling.

Timing and location of capsid disassembly are not well characterized and may differ depending on the target cell type. Currently, there are two main models for HIV-1 uncoating: (i) gradual capsid dissociation within the cytosol during reverse transcription and transport to the NPC and (ii) reverse transcription in intact or largely intact capsids, followed by their dissociation at the NPC (reviewed in references 12 and 13). Novel tools for investigating the integrity of individual postfusion complexes using fluorescence microscopy of living cells were recently reported, but the exact time and mechanism of uncoating are still not clear. Monitoring of postfusion subviral particles using intravirion fluid-phase markers indicated rapid reverse transcription-induced loss of capsid integrity approximately 30 min after fusion in cultured cell lines, primary T cells, and macrophages (18). On the other hand, live cell imaging of single HIV-1 complexes using incorporated cyclophilin A-DsRed as a surrogate capsid marker suggested loss of CA from subviral complexes at the NPC in HeLa-derived TZM-bl cells, while earlier uncoating in the cytoplasm appeared to promote proteasomal degradation of viral complexes (19).

Variable amounts of CA have been detected on nuclear HIV-1 PICs in different cell types (20–27). It is currently not clear whether CA colocalizing with nuclear subviral complexes represents residual CA molecules remaining on the nucleoprotein complex or whether CA lattice architecture is retained, at least in part. Furthermore, the role of nuclear CA (if any) is currently unknown. A recent study suggested bimodal binding of the host cell protein NONO to the capsid lattice and the DNA sensor cGAS, thereby facilitating HIV-1 genome sensing in the nucleus (28). CA and the hexameric CA lattice have been shown to bind to several nucleoporins and to the host cell protein cleavage and polyadenylation specificity factor 6 (CPSF6) (29–34), suggesting a role of the capsid lattice in transport to and/or entry into the nucleus (reviewed in references 12 and 35). In primary human macrophages, CPSF6 strongly accumulates on nuclear PICs in a CA-dependent manner, and lack of CPSF6 binding (by alteration of the binding site in CA or depletion of CPSF6) led to arrest of HIV-1 subviral complexes directly at the NPC (27). Another recent study showed that CA-CPSF6 interaction was required for nucleoplasmic entry of the HIV-1 PIC with CPSF6 binding-defective variants retaining subviral complexes in the nuclear periphery of infected macrophages (36). These observations indicate that, at least in macrophages, CPSF6 plays an important role for nuclear entry of HIV-1 replication complexes through its interaction with the CA lattice. On the other hand, CPSF6 and its binding to CA appear to be dispensable for HIV-1 infectivity in T cells (36, 37), while CPSF6 was reported to be involved in targeting HIV-1 integration in this cell type (36). The role of CPSF6 for nuclear entry and nucleoplasmic trafficking in T cells (if any) is currently not clear, however.

Here, we developed an experimental system that allows discrimination of HIV-1 postfusion complexes inside the cytoplasm from virions at the plasma membrane or in endosomes of the T-cell line SupT1-R5. This imaging-based system permits robust identification of postfusion subviral structures and analysis of their content and association with host cell factors over the background of viruses trapped in endosomes or at the plasma membrane. Following characterization of HIV-1 early replication kinetics in this cell line, we analyzed the CA content of HIV-1 subviral complexes at different subcellular sites. Our analysis indicated loss of free CA protein from postfusion HIV-1 complexes, while the CA content of the assembled lattice appeared to remain constant until the complexes reached the nuclear pore. Nuclear PICs were largely devoid of detectable CA. An HIV-1 variant with defective CPSF6-binding exhibited accumulation of CA-containing subviral complexes directly at the NPC, similar to its phenotype in macrophages. These data suggest that CPSF6 also plays a role in nucleoplasmic entry of HIV-1 replication complexes in T cells while not being required for infectivity in this cell type.

## RESULTS

### Kinetics of HIV-1 entry and early replication in SupT1-R5 cells.

We used the CD4^+^ T-cell line SupT1-R5 for this study, since these cells are highly susceptible to infection by X4- and R5-tropic strains of HIV-1, express high levels of CD4, similar to activated primary T cells, and show little unspecific virus attachment, with HIV-1 binding being largely CD4 dependent and occurring on CD4 clusters (38, 39). As described previously (40), we synchronized virus entry by adsorption of particles for 90 min at 16°C, where neither membrane fusion (41, 42) nor endocytosis (43, 44) occurs. After removal of the inoculum, virus entry was initiated by temperature shift to 37°C. To characterize kinetics of early HIV-1 replication as a baseline for our imaging experiments, inhibitors of different steps of HIV-1 replication were subsequently added at various time points. Infection was scored 48 h postinfection (p.i.) by determining the proportion of CA-positive cells via flow cytometry.

**FIG 1:**
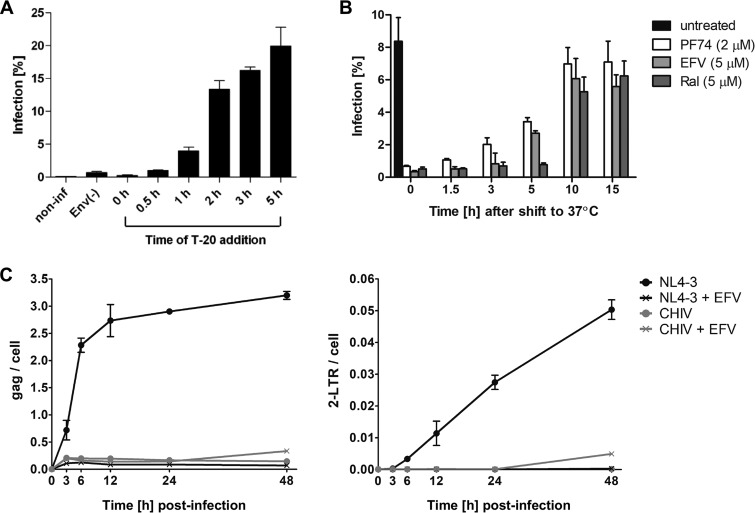
Kinetics of productive HIV-1 entry into SupT1-R5 cells. (A and B) Cells were incubated with HIV-1_NL4-3_ for 90 min at 16°C. After adsorption, inoculum was changed for fresh medium and cells were shifted to 37°C to initiate virus entry. At the indicated time points after temperature shift, T-20 (A) or PF74, EFV, or Ral (B) was added. Infection was scored at 48 h p.i. by immunostaining for intracellular HIV-1 CA protein followed by flow cytometry. Noninfected cells, cells infected with equal amounts of HIV-1_NL4-3_ lacking Env glycoprotein [Env(−)], and untreated cells were used as controls. Bars indicate mean values and standard deviations (SD) from one representative experiment performed in triplicate. (C) Quantitation of reverse transcription (RT) products using ddPCR. Cells were infected with HIV-1_NL4-3_ or non-replication-competent HIV-1_CHIV_ particles and harvested at the indicated time points, and HIV-1 RT products were quantitated in cell lysates by ddPCR. Copy numbers of late RT products (C, left) or 2-LTR (C, right) circles were normalized to the copy numbers of the housekeeping gene RPP30. Graphs show mean values and SD from triplicates.

To define the period during which most fusion events occurred in SupT1-R5 cells, we employed the fusion inhibitor T-20 (Fig. 1A). Infection was almost abolished when T-20 was added 0 to 30 min after temperature shift, indicating that only a few productive entry events had occurred during this time period. Infection became gradually refractory to inhibition by T-20 over the observation period from 1 to 5 h after temperature shift. The steepest increase in productive entry events occurred between 1 and 2 h after the temperature shift, defining a time frame for optimal detection of cytosolic subviral complexes. The time course of early postfusion events was followed by adding specific HIV-1 inhibitors targeting either reverse transcription (using the nonnucleosidic RT inhibitor efavirenz [EFV]) or genome integration (using the IN inhibitor raltegravir [RAL]). Very low infection rates were observed when EFV was added up to 3 h after the temperature shift, and ∼50% inhibition was observed when EFV was added at 5 h (Fig. 1B). Virus infection was still fully sensitive to RAL at this time point and became resistant to both inhibitors at 10 h p.i. (Fig. 1B). PF74, targeting the HIV-1 capsid lattice, has been suggested to act as an inhibitor of nuclear import at 2 μM concentration (21, 45, 46) and was used here in parallel. PF74 exhibited an inhibitory profile similar to that of EFV but already lost some activity when added at 3 h p.i. (Fig. 1B).

Inhibitor time-of-addition experiments were complemented by analysis of HIV-1-specific RT products using digital droplet PCR (ddPCR) (47). SupT1-R5 cells were infected with wild-type HIV-1 or a replication-incompetent derivative lacking the viral long terminal repeats (LTR) (CHIV [48]) in the presence or absence of EFV. HIV-specific RT products were quantitated using primers detecting the viral *gag* gene or 2-LTR circles, the latter being a surrogate marker for HIV-1 cDNA imported into the nucleus (49) (Fig. 1C). Late RT products were detected from 3 h p.i. onwards for wild-type HIV-1 and reached a plateau at 12 h (Fig. 1C, left); the majority of late RT products were synthesized between 3 and 6 h p.i. 2-LTR circles were detected from 6 h p.i. onwards and accumulated with linear kinetics until the end of the observation period (48 h p.i.; Fig. 1C, right). These results were in line with the inhibitor time-of-addition experiments and confirmed that reverse transcription in SupT1-R5 cells occurs with a time course similar to that reported for lymphoid cells (50). No specific ddPCR products were detected upon infection in the presence of EFV or with the replication-incompetent variant lacking the viral LTR (Fig. 1C).

### Establishing mCLING as membrane marker for the study of HIV-1 cytosolic entry into SupT1-R5 cells.

In order to distinguish cytoplasmic postfusion complexes from virions at the plasma membrane or within endosomes, we established labeling of infected cells with the membrane-binding fluorophore cysteine lysine palmitoyl group (mCLING [51]). This compound has been shown to be stably incorporated into the plasma membrane and is retained on endosomal vesicles that have been internalized after addition of the compound (51). It remains attached to membranes upon fixation and cell permeabilization and therefore can be used in combination with immunostaining (51). Therefore, applying mCLING to cells after virus adsorption should allow distinguishing HIV-1 particles at the plasma membrane or within endosomes (mCLING^+^) from postfusion complexes that have entered the cytoplasm (mCLING^−^).

By titration experiments, we established that a final concentration of 2 μM mCLING.Atto647N ensured strong staining of SupT1-R5 cell membranes, and this concentration was subsequently used for all imaging experiments. Possible cytotoxic effects of mCLING staining were assessed by measuring viability of SupT1-R5 cells stained with the probe for 15 h at 37°C (the longest time point in our time-of-addition experiments; Fig. 1B). Viability was determined either directly or after an additional 48 h of cultivation in the absence of mCLING. mCLING concentrations up to 5 μM did not affect cell viability in either case (see Fig. S1 in the supplemental material).

10.1128/mBio.02501-19.1 FIG S1 Effect of mCLING labeling on viability of SupT1-R5 cells. (A and B) Cells were incubated in the presence of the indicated concentrations of mCLING.Atto647N for 15 h at 37°C. Viability was assessed by trypan blue exclusion using the Countess automated cell counter either directly (A) or upon further incubation for 48 h in the absence of mCLING (B). Values for 2 μM mCLING (concentration used in microscopy experiments) are highlighted in red. Download FIG S1, TIF file, 0.5 MB. Copyright © 2019 Zila et al. 2019 Zila et al. This content is distributed under the terms of the Creative Commons Attribution 4.0 International license.

**FIG 2:**
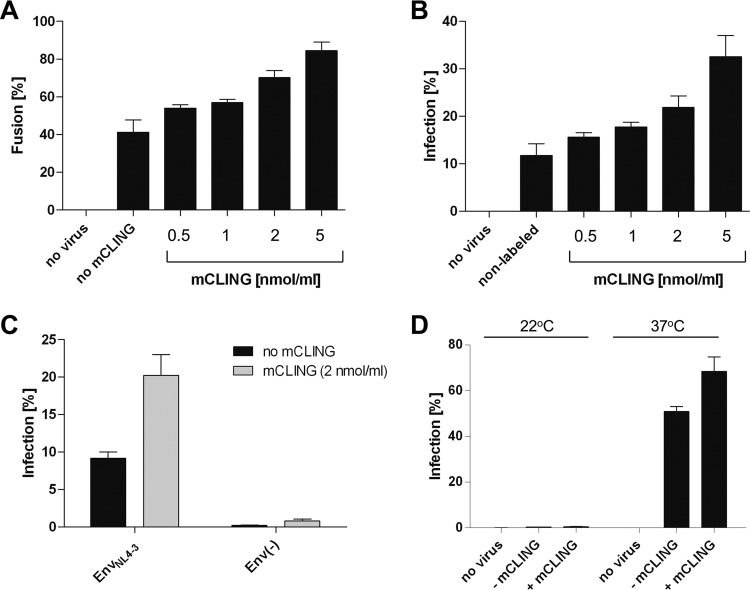
Effect of mCLING labeling on HIV-1 entry efficiency. (A and B) SupT1-R5 cells were incubated with HIV-1_NL4-3_ Vpr.BlaM (0.2 μU of RT/cell) (A) or HIV-1_NL4-3_ (2.8 μU of RT/cell) (B) for 120 min at 16°C. After adsorption, mCLING.Atto647N was added at the indicated concentration, and cells were incubated for an additional 10 min at 16°C and subsequently shifted to 37°C to initiate virus entry. (A) After 2 h at 37°C, cells were washed and processed to determine cytosolic entry efficiency using the BlaM assay. (B) Alternatively, T-20 was added after 2 h at 37°C, and cells were further incubated at 37°C and fixed 48 h after the shift to score infectivity. Noninfected cells and cells infected in the absence of the probe were used as controls. (C) Test for unspecific HIV-1 cytosolic entry via mCLING-mediated membrane fusion. SupT1-R5 cells were incubated with HIV-1_NL4-3_ lacking or carrying Env (both at 2.8 μU RT/cell) for 90 min at 16°C. Cells were then stained with 2 μM mCLING.Atto647N for 10 min at 16°C and shifted to 37°C. T-20 was added after 2 h, and incubation was continued for 46 h before scoring infected cells by flow cytometry. (D) Contribution of endocytosis to HIV-1 infection in the presence of mCLING. SupT1-R5 cells were preincubated for 10 min at 22°C in the absence or presence of mCLING.Atto647N (2 μM). Subsequently, HIV-1_NL4-3_ (6.9 μU of RT/cell) was added and cells were incubated for 4 h at 22°C to allow for endocytosis, but not fusion, prior to addition of T-20 to inhibit any fusion occurring from the plasma membrane. In parallel, infected cells were incubated for 4 h at 37°C as virus entry control. Cells were incubated at 22°C for one more hour and subsequently shifted to 37°C. To determine infection efficiency, cells were fixed at 48 h p.i., immunostained for intracellular CA, and scored using flow cytometry. The graph shows mean values and SD from one representative experiment performed in triplicate.

The experimental conditions used also resulted in staining of viral envelopes, albeit less efficient than that of cellular membranes. Attachment of mCLING to the virion, and/or to the plasma membrane, might affect infectivity of the virus. To determine whether mCLING has an effect on HIV-1 cell entry or viral replication, we compared HIV-1 membrane fusion and infectivity in the presence or absence of the compound. To follow cytosolic entry, we employed an established HIV-1 entry assay detecting the cytosolic release of β-lactamase (BlaM) incorporated into viral particles as a Vpr.BlaM fusion protein (52). Cells were preincubated with Vpr.BlaM containing HIV-1 at 16°C, stained with various mCLING concentrations for an additional 10 min at 16°C, and shifted to 37°C for 2 h prior to processing for the fusion assay. Productive entry was analyzed in cells stained in parallel with mCLING and infected with wild-type HIV-1. At 2 h p.i., T-20 was added to block further entry and second-round infections, and the proportion of infected cells was scored at 48 h p.i. Treatment with mCLING clearly did not inhibit either HIV-1 entry or viral replication. In contrast, it increased entry efficiency and infectivity in a dose-dependent and parallel manner (Fig. 2A and B), indicating a stimulatory effect on virus binding and/or membrane fusion. To determine possible effects on virus binding, cells were prestained with mCLING for 1 h at 16°C; subsequently virus was added and cells were incubated for an additional 1 h at 16°C. Cells were then fixed and subjected to HIV-1 CA immunostaining and confocal microscopy. Quantification of cell-associated particles from maximal z-projections of total cell volumes showed no significant difference in virus binding between unstained and mCLING-stained cells (Fig. S2), suggesting that the observed enhancement of entry and infectivity upon mCLING staining was due to increased membrane fusion. At 2 μM concentration, mCLING stimulated viral entry and infection by a factor of 1.7 and 1.9, respectively.

10.1128/mBio.02501-19.2 FIG S2 mCLING does not affect binding of HIV-1 to cells. (A and B) SupT1-R5 cells were incubated in the absence or presence of mCLING.Atto647N (2 μM) for 60 min at 16°C. Subsequently, HIV-1_NL4-3_ carrying or lacking Env (6.4 μU of RT/cell) was added and cells were incubated for an additional 90 min at 16°C. Cells were washed and transferred to PEI-coated 8-well chamber slides, fixed, permeabilized, and immunostained for HIV-1 CA protein. (A) Numbers of particles associated with the cell surface were determined from the maximum-intensity projections of z-stacks as shown in panel B using the Icy software spot detection function (B, lower right; yellow encircled HIV-1 CA signals in green regions of interest). The graph shows mean values and SEM from *n* cells from four randomly selected optical fields. Download FIG S2, TIF file, 1.9 MB. Copyright © 2019 Zila et al. 2019 Zila et al. This content is distributed under the terms of the Creative Commons Attribution 4.0 International license.

To test whether incorporation of mCLING into the virion envelope and/or cell membrane could promote HIV-1 replication independently of the Env glycoprotein, we infected SupT1-R5 cells with HIV-1 particles carrying or lacking Env and scored for infected cells as described above. No Env-independent infection was observed upon mCLING staining (Fig. 2C).

We have previously shown that endocytosis contributes little to productive HIV-1 entry in SupT1-R5 and primary CD4^+^ T cells (40). Since mCLING has been described as an endocytic marker (51), we wanted to test whether mCLING can redirect productive virus entry to the endosomal route. For this, we employed a previously described temperature shift experiment (40). Cells were incubated with infectious HIV-1 for 5 h at 22°C (a temperature that blocks HIV-1 fusion but allows for endocytosis) in the presence or absence of mCLING, followed by addition of T-20 and shift to 37°C. The peptidic compound T-20 cannot reach virions internalized into endosomes during room temperature preincubation but will block viral entry from the plasma membrane. Infectivity was scored 48 h after temperature shift. No residual infectivity was detected when T-20 was added 5 h after incubation at 22°C (Fig. 2D), although substantial endocytosis of HIV-1 occurs at this temperature (40). We conclude that the observed increase in HIV-1 cell entry and infectivity upon mCLING treatment was not due to increased virus binding, enhanced unspecific fusion, or productive endocytic entry but rather reflected more efficient receptor-mediated fusion of the viral and plasma membranes.

**FIG 3:**
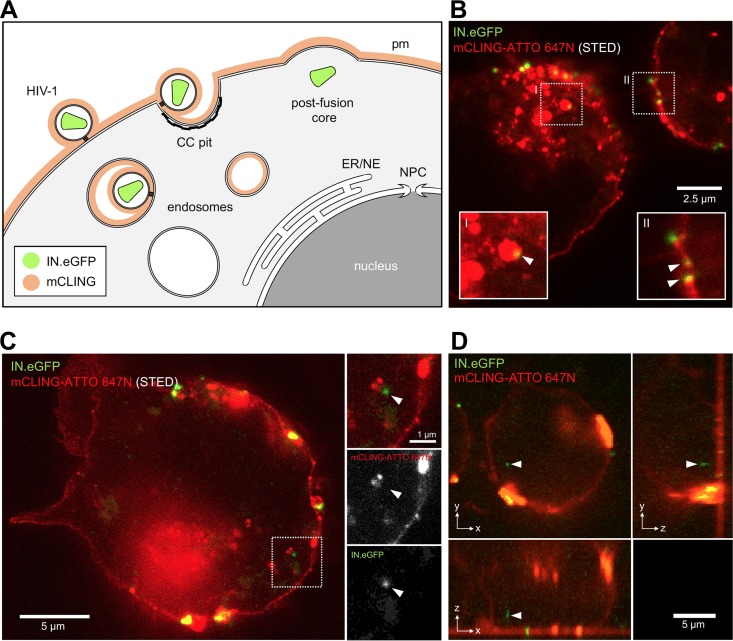
Approach to identify HIV-1 postfusion complexes. (A) Application of mCLING to distinguish postfusion HIV-1 complexes from complete virions at the plasma membrane (PM) or within endosomes. PM and endosomes are stained by mCLING, whereas membrane-less viral complexes lack the mCLING signal. PM, plasma membrane; CC, clathrin-coated pit; ER, endoplasmic reticulum; NE, nuclear envelope; NPC, nuclear pore complex. (B to D) SupT1-R5 cells were incubated with IN.eGFP-carrying HIV-1_CHIV_ particles (0.5 μU of RT/cell) for 90 min at 16°C. Cells were then stained with mCLING.Atto647N for an additional 10 min at 16°C and shifted to 37°C to initiate virus entry. Cells were fixed with 4% FA–0.2% GA at 30 min (D) or 2 h (B and C) after temperature shift. IN.eGFP signals (green) were readily detected in association with mCLING-labeled (red) endosomes (B, I) or plasma membrane (B, II) and also inside the cytoplasm of cells without colocalization with mCLING (C and D). In panels B and C, STED microscopy was used to visualize the mCLING signal. White arrowheads point to the position of the selected IN.eGFP signals.

We then followed HIV-1 entry into mCLING-labeled SupT1-R5 cells in order to identify postfusion complexes. We used HIV-1 particles carrying IN.eGFP (a marker for complete viral particles as well as for RTCs/PICs [53]); these particles were previously shown to exhibit infectivity similar to that of wild-type HIV-1 (21). Cells were incubated with virus particles for 90 min at 16°C to allow for plasma membrane attachment and subsequently stained with mCLING.Atto647N for an additional 10 min at 16°C. This procedure ensured efficient incorporation of mCLING into the plasma membrane (Fig. 3 and Fig. S3). The temperature was then shifted to 37°C. Cells were fixed at various time points after temperature shift and examined by confocal and stimulated emission depletion (STED) microscopy. mCLING staining was restricted to the plasma membrane at the time of temperature shift, whereas numerous mCLING-positive endosomal vesicles were detected when samples were fixed 30 min later (Fig. S3). Some of these mCLING-positive endosomal structures colocalized with IN.eGFP signals (Fig. 3B and Fig. S3), indicating virus endocytosis during the incubation period. Importantly, we also observed intracellular IN.eGFP-labeled complexes that were mCLING negative after the temperature shift (Fig. 3C and D), indicative of postfusion HIV-1 complexes.

10.1128/mBio.02501-19.3 FIG S3 Endocytic uptake of mCLING during synchronized HIV-1 entry. SupT1-R5 cells were incubated with IN.eGFP-carrying HIV-1_CHIV_ particles (1.6 μU of RT/cell) for 90 min at 16°C. After adsorption, cells were transferred to PEI-coated 8-well chamber slides and stained with mCLING.Atto647N for 10 min at 16°C. Samples were shifted to 37°C for the indicated times, fixed, and imaged by spinning disk confocal microscopy. Images show confocal sections. Arrowheads in enlargements indicate IN.eGFP-labeled virions at the plasma membrane (i) or in endosomes (ii). Download FIG S3, TIF file, 1.9 MB. Copyright © 2019 Zila et al. 2019 Zila et al. This content is distributed under the terms of the Creative Commons Attribution 4.0 International license.

An Env-deficient virus variant was used to validate whether intracellular IN.eGFP-positive and mCLING-negative complexes truly represent postfusion complexes rather than virus particles within insufficiently labeled endosomes. For this, we examined SupT1-R5 cells infected with IN.eGFP-labeled HIV-1 particles pseudotyped with either the wild type or a fusion-incompetent Env variant (40). Virus entry was synchronized through prebinding at 16°C, followed by mCLING staining and subsequent shift to 37°C. Cells were fixed at 90 min after the temperature shift, at the peak of fusion activity in our experimental setup (Fig. 1A). HIV-1 postfusion complexes are expected to constitute relatively rare events over a large background of nonfused virions at the plasma membrane and within endosomes even for wild-type virus.

**FIG 4:**
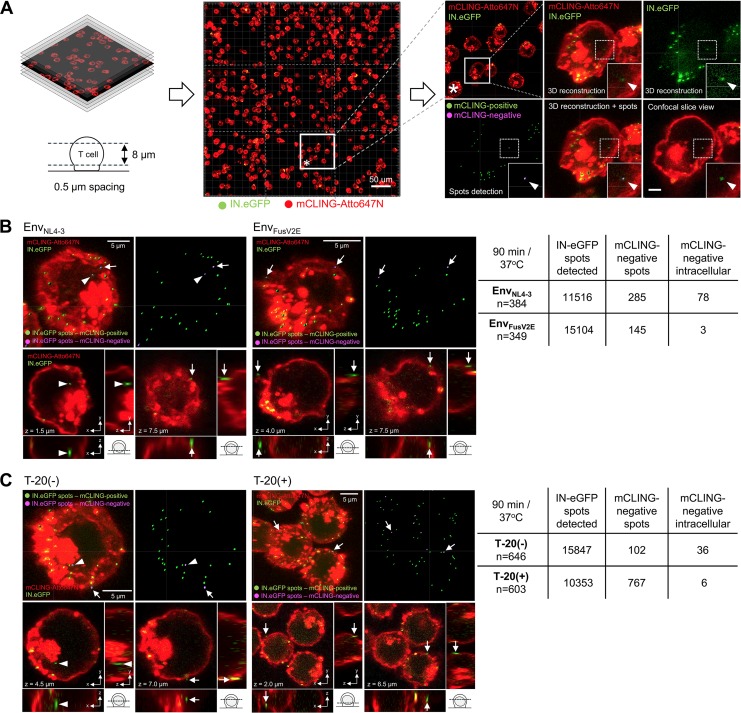
Validation of mCLING-based detection of HIV-1 postfusion complexes. (A) Experimental workflow. SupT1-R5 cells were infected with IN.eGFP-labeled HIV-1 particles in the presence of mCLING. Z-stacks were acquired using confocal microscopy and merged to cover a section of 8 μm through the cell body. 3D volumes were reconstructed from these image series and analyzed using the Imaris spot detection function that creates a 3D ellipsoid object (lower right, green) for each recognized IN.eGFP signal (upper right). Objects were classified as intracellular by manual assignment based on the 3D data. Intracellular objects displaying an mCLING signal intensity below the threshold (lower right, violet; see Materials and Methods) were classified as postfusion HIV-1 complexes. See also Movie S1 in the supplemental material. (B) Cells were incubated with IN.eGFP-labeled HIV-1_CHIV_ particles pseudotyped with wild-type Env glycoprotein (Env_NL4-3_) or with the fusion-incompetent Env mutant (EnvFusV2E) (both at 1.6 μU RT/cell) for 90 min at 16°C. After adsorption, cells were stained with mCLING.Atto647N (2 μM) for 10 min at 16°C and then shifted to 37°C for 90 min. Cells were transferred to 8-well chamber slides, fixed with 4% FA–0.2% GA, and analyzed as outlined for panel A. (C) Cells were incubated with IN.eGFP-carrying HIV-1_CHIV_ particles (0.6 μU RT/cell) for 90 min at 16°C. Incubation was continued in the absence or presence of T-20 for an additional 30 min at 16°C prior to shift to 37°C for 90 min. Cells were processed and fixed as described for panel B. (B and C) Top panels show 3D reconstructions of representative cells with IN.eGFP signals either colocalizing (green dots) or not colocalizing (violet dots) with mCLING. Bottom panels show selected IN.eGFP signals in corresponding confocal slices with corresponding orthogonal views. Arrowheads and arrows point to selected intracellular or extracellular mCLING-negative IN.eGFP signals, respectively. Results from quantitation are summarized in the tables on the right.

In order to obtain statistically relevant and unbiased data, we established an automated workflow for microscopy acquisition of z-stacks of large (0.5 mm by 0.5 mm) areas containing ∼350 to 650 cells each. Three-dimensional (3D) volume reconstruction of the cell bodies was followed by automated detection and analysis of cell-associated viral objects. Detected objects were manually classified as intra- or extracellular based on the 3D data; intracellular IN.eGFP^+^/mCLING^−^ objects were considered to represent postfusion complexes (Fig. 4A). This semiautomated workflow allowed us to evaluate >10,000 HIV-1-specific structures per condition. In the case of virus carrying wild-type Env, a total of 78 IN.eGFP^+^, mCLING.Atto647N^−^ structures were detected within the cells, corresponding to 0.7% of all IN.eGFP-positive structures analyzed (Fig. 4B). In contrast, only three such complexes (0.02% of all IN.eGFP-positive complexes; Fig. 4B) were observed in the case of the virus carrying the fusion-incompetent Env variant.

10.1128/mBio.02501-19.7 MOVIE S1 Workflow for mCLING-based identification of HIV-1 postfusion complexes. SupT1-R5 cells were infected with IN.eGFP-carrying HIV-1_NL4-3_ (green) in the presence of mCLING.Atto647N (red). Z-stacks were acquired and analyzed for colocalization of IN.eGFP with mCLING.Atto647N. Step 1, the application of Imaris spot detection function creates a 3D ellipsoid object for each recognized individual IN.eGFP signal. Step 2, for each object, the signal in the mCLING channel is measured. Objects with an mCLING signal below the threshold (see Materials and Methods) are classified as mCLING negative (violet). Step 3, violet objects located within the cell interior are identified as postfusion HIV-1 complexes. Download Movie S1, AVI file, 11.6 MB. Copyright © 2019 Zila et al. 2019 Zila et al. This content is distributed under the terms of the Creative Commons Attribution 4.0 International license.

These findings were supported by infection in the presence or absence of 50 μM T-20 (Fig. 4C). In the absence of the fusion inhibitor, we observed 36 IN.eGFP^+^, mCLING.Atto647N^−^ complexes (0.24% of all IN.eGFP^+^ complexes), while only 6 (0.07%) of such complexes were counted in the presence of T-20. Taken together, these data indicate that staining of cells with preadsorbed virus with mCLING.Atto647N prior to synchronized virus entry, combined with a fluorescent marker for the virus, is a straightforward strategy for robust detection of HIV-1 postfusion complexes.

### Presence of CA on HIV-1 postfusion complexes at different subcellular localizations in SupT1-R5 cells.

Using the validated approach for detection of postfusion complexes, we proceeded to analyze the association of CA with HIV-1 subviral complexes at different subcellular localizations. For this, we performed CA immunostaining of cells infected with IN.eGFP-carrying virus at various times postentry. The observed complexes were then classified according to their subcellular localization (cytoplasmic, NPC, or nuclear).

**FIG 5:**
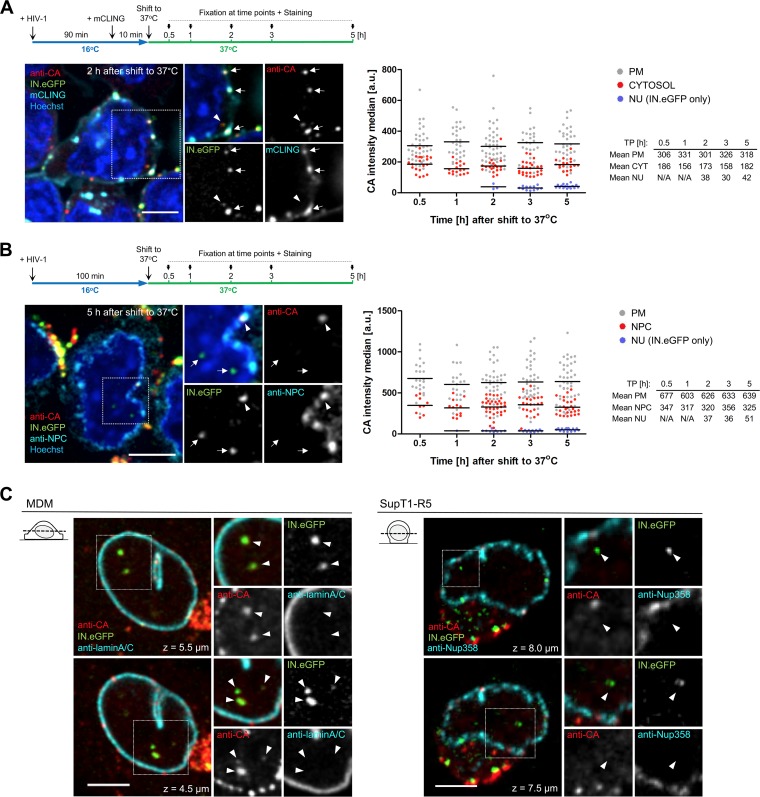
CA content of subviral HIV-1 complexes drops by 50% after fusion and appears to be lost upon nucleoplasmic entry. (A and B) SupT1-R5 cells were incubated with IN.eGFP-carrying HIV-1_NL4-3_ virions (2 μU of RT/cell) for 90 min at 16°C. To visualize postfusion HIV-1 complexes, cells were stained with mCLING.Atto647N for 10 min at 16°C. Subsequently, the inoculum was changed for medium with (A) or without (B) mCLING, and cells were transferred to PEI-coated 8-well chamber slides, shifted to 37°C for the indicated time, and fixed with 4% FA–0.2% GA (A) or 4% FA (B). See schematic illustrations of the experiment above fluorescent panels. Samples were immunostained for HIV-1 CA (A and B) and NPC (B). DNA was stained with Hoechst. Confocal sections of cells with enlarged details are shown. In panel A, the arrowhead indicates an mCLING-negative HIV-1 complex inside the cytosol, and arrows indicate HIV-1 virions at the plasma membrane. In panel B, the arrowhead indicates a viral complex at the NPC and arrows point to IN.eGFP-positive complexes in the nucleus. Scale bars, 5 μm. (A and B) The graphs on the right show distribution and intensities of CA signals detected at the indicated time points (TP) for virions at the plasma membrane (PM), subviral complexes in the cytosol (A) or in association with the NPC (B), and for IN.eGFP-labeled complexes inside the nucleus (NU). 3D reconstructions based on z-stacks of cells were analyzed using the Imaris spot detection function applied on the CA and IN.eGFP signals. The median of CA signal intensity in the detected objects was then measured. For intranuclear complexes, only IN.eGFP spots were analyzed. At least 40 cells were analyzed per TP. CA intensities were lower in mCLING-stained samples (A, right), possibly due to a negative effect of the required treatment with 0.2% glutaraldehyde (51) on antigenicity. a.u., arbitrary units; N/A, not applicable. (C) Detection of CA on nuclear HIV-1 complexes after copper-based extraction of infected cells. MDM or SupT1-R5 cells were infected with R5-tropic, IN.eGFP-carrying HIV-1_NL4-3_ (6.7 × 10^5^ μU of RT/well) at 37°C for 48 h or 5.5 h, respectively. Samples were fixed with 4% FA in PBS, permeabilized with 0.5% Triton X-100, and then extracted using the Click-IT reaction mixture usually employed for copper-catalyzed EdU click labeling. Cells were immunostained for HIV-1 CA and lamin A/C (MDM) (left) or Nup358 (SupT1-R5) (right). Z-sections through the nuclear regions of representative cells are shown. Arrowheads indicate IN.eGFP-positive viral complexes inside the nucleus.

Parallel cultures of SupT1-R5 cells were preincubated with IN.eGFP-labeled virions for 90 min at 16°C and were either left untreated or stained with mCLING.Atto647N for an additional 10 min at 16°C before shifting the temperature to 37°C (Fig. 5). Cells infected in the presence of mCLING.Atto647N were immunostained only with antiserum against HIV-1 CA; cells infected in the absence of the probe were immunostained in addition with an antibody against NPC proteins (FG repeats) to identify subviral complexes localized at the nuclear envelope. IN.eGFP^+^ complexes were identified in 3D volume reconstructions of infected cells and analyzed for CA signal intensity and subcellular location (Fig. 5). Complexes were classified as being located (i) at the plasma membrane, (ii) within the cytosol (mCLING-stained samples) or at the nuclear envelope (samples stained for FG repeats), and (iii) inside the nucleus.

For all samples, the mean CA signal intensity associated with complexes in the cytosol (Fig. 5A, right) or close to the nuclear envelope (Fig. 5B, right) was ∼50% lower than the CA intensity of virions attached to the plasma membrane in the same experiment. In contrast to our previous observations in monocyte-derived primary macrophages (MDM [21, 27]), IN.eGFP^+^ complexes located inside the nucleus of SupT1-R5 cells appeared to be devoid of a detectable CA signal (Fig. 5A and B).

In order to ensure that the observed cell type-dependent difference was not due to slightly different detection conditions, we subjected SupT1-R5 cells and MDM to parallel infections and used the identical procedure for detection of subviral complexes (21, 26, 27) (Fig. 5C). SupT1-R5 cells and MDM were infected with HIV-1 IN.eGFP particles pseudotyped with an R5-tropic Env protein (54) at 37°C for 5.5 h or 48 h, respectively. After fixation, cells were treated with the Click-IT reaction mixture that we had employed for copper-catalyzed 5-ethynyl-2′-deoxyuridine (EdU) click labeling in our former studies, followed by detection of intranuclear subviral complexes by immunostaining with an antiserum against HIV-1 CA. The nuclear envelope was visualized by immunostaining of lamin A/C (MDM) or Nup358 (SupT1-R5). At 48 h p.i., CA was detected on the vast majority of nuclear HIV-1 complexes in MDM (92.5%), in accordance with our previous observations (21, 26, 27). In contrast, nuclear IN.eGFP^+^ complexes in SupT1-R5 cells appeared to be largely devoid of CA under these modified conditions (Fig. 5C), with only 4.5% of these subviral complexes exhibiting a weak CA signal. Taken together, these findings suggest that HIV-1 capsids entering the cytosol retain ca. 50% of virion-associated CA throughout their journey through the cytoplasm, and this CA signal remains stable when the subviral complexes reach the nuclear envelope. The vast majority of CA appears to be lost upon nucleoplasmic entry of subviral HIV-1 complexes in SupT1-R5 cells, while the CA signal remains largely unchanged on nuclear HIV-1 complexes in MDM. To examine the effect of RT inhibition on uncoating and nuclear import of HIV-1 complexes in SupT1-R5 cells, cells were infected for 5 h in the absence or presence of EFV, followed by immunostaining against HIV-1 CA. Nuclear import of IN.eGFP-positive complexes was independent of reverse transcription (Fig. S4), similar to previous observations in MDM and HeLa cells (27, 55).

10.1128/mBio.02501-19.4 FIG S4 Influence of reverse transcription on HIV-1 nuclear import. (A) SupT1-R5 cells were incubated with IN.eGFP-carrying HIV-1_NL4-3_ virions (2 μU of RT/cell) for 90 min at 16°C. After adsorption, EFV (5 μM) or DMSO only (control) was added and cells were transferred to PEI-coated 8-well chamber slides and shifted to 37°C for 5 h. Samples were immunostained for HIV-1 CA (red) and NPC (cyan). DNA was stained with Hoechst. (B) Number of nuclear IN.eGFP-labeled complexes in cells infected in the presence of DMSO or EFV, determined from images as shown in panel A. Mean values and SEM for *n* cells from at least 3 tiled optical fields (3 by 3) stitched together (representing an area of 0.5 mm^2^) are shown. Download FIG S4, TIF file, 2.2 MB. Copyright © 2019 Zila et al. 2019 Zila et al. This content is distributed under the terms of the Creative Commons Attribution 4.0 International license.

### The role of CPSF6 in nuclear import of subviral HIV-1 complexes in SupT1-R5 cells.

The host protein CPSF6 interacts with the HIV-1 CA lattice and has been implicated in different stages of early replication (13). In MDM, depletion of CPSF6 or impairment of CPSF6-CA interaction resulted in pronounced retention of HIV-1 PICs at the NPC, accompanied by a modest reduction of HIV-1 infectivity (27). Detectable but moderate impairment of infectivity caused by CPSF6 depletion or disturbance of CA-CPSF6 interaction through CA mutation N74D had also been demonstrated in earlier studies on macrophages (56, 57).

**FIG 6:**
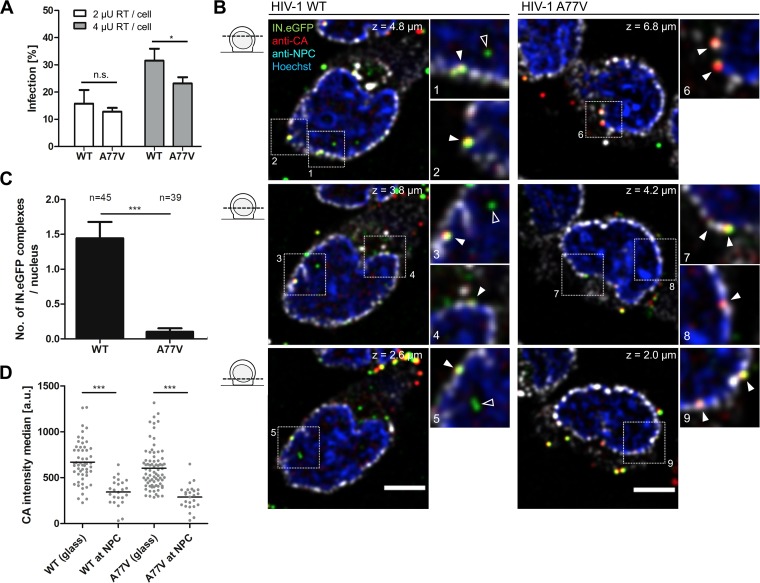
CPSF6 binding-defective mutant A77V accumulates at the NPC without major loss of infectivity. (A) SupT1-R5 cells were infected with the indicated amounts of HIV-1_NL4-3_ wild type (WT) or A77V. At 48 h p.i., cells were immunostained for intracellular HIV-1 CA and infection was scored by flow cytometry. The graph shows mean values and SD from three independent experiments performed in triplicates. (B) SupT1-R5 cells were incubated with IN.eGFP-carrying HIV-1_NL4-3_ WT or A77V virions (2 μU of RT/cell) for 90 min at 16°C. After adsorption, inoculum was removed and cells were shifted to 37°C to initiate virus entry. Cells were fixed 8 h after temperature shift and immunostained for HIV-1 CA and NPC. DNA was stained with Hoechst. Deconvolved confocal sections through the nuclear region of representative cells infected with WT or A77V HIV-1 are shown. Arrowheads in enlargements indicate CA^+^ and IN.eGFP^+^ complexes associated with NPC (filled arrowheads) and IN-eGFP^+^ complexes inside the nucleus (open arrowheads). Scale bars, 5 μm. See Fig. S5 for enlarged regions 1 to 9 separated by fluorescence channels. (C) Numbers of IN.eGFP^+^ complexes in nuclei of HIV-1 WT- or A77V-infected cells. Mean values and standard errors of the means (SEM) for *n* cells from at least 8 randomly chosen fields of view are shown. (D) Distribution and intensities of CA signals detected 8 h after the temperature shift for WT or A77V extracellular virions (adhered to the 8-well chamber slides) or subviral complexes associated with the NPC. Z-stacks of cells were analyzed using the Imaris spot detector applied to the IN.eGFP signals, and the median of CA signal intensity of these objects was determined as described in Materials and Methods. Objects in more than 10 cells per condition were analyzed. Mean intensity values are indicated by black lines. Statistical significance was assessed by a nonpaired two-tailed Student's *t* test. *, *P* < 0.05; ***, *P* < 0.0001; n.s., not significant.

To test whether a similar phenotype is also observed in SupT1-R5 cells, we first analyzed the effect of CPSF6 binding deficiency on HIV-1 infectivity. Cells were infected with two different amounts of either wild-type HIV-1 or a variant deficient in CPSF6 binding (A77V). T-20 was added at 5 h p.i. to prevent secondary rounds of infection, and the proportion of infected cells was scored at 48 h p.i. by CA immunostaining and flow cytometry. As shown in Fig. 6A, infectivity was mildly reduced for the A77V variant compared to the wild type. We then tested whether infection by the A77V variant required nuclear envelope breakdown in SupT1-R5 cells. Treatment of cells with 1 μM aphidicolin (APC) starting 16 h prior to infection led to a complete block in cell proliferation (Fig. S6B) and a dramatic reduction in chromosomal DNA replication (Fig. S6C), indicating complete cell cycle arrest. HIV-1 infection in APC-arrested cells was mildly and similarly reduced for both the wild type and the A77V variant (Fig. S6A), suggesting that infection by the CPSF6 binding-defective A77V variant does not require nuclear envelope breakdown.

10.1128/mBio.02501-19.6 FIG S6 Infectivity of CPSF6 binding-defective HIV-1 mutant in cell cycle-arrested cells. (A) SupT1-R5 cells were pretreated with APC (1 μM) for 16 h at 37°C and then infected with HIV-1 wild type (WT) or A77V in the presence of the drug. After 24 h, the inoculum was replaced by fresh medium supplemented with 50 μM T-20 and APC. At 48 h p.i., cells were fixed and immunostained for intracellular HIV-1 CA. Infection was scored by flow cytometry. As controls, cells pretreated and infected in the presence of DMSO and noninfected cells were used. The graph shows mean values and SD from three independent experiments performed in quadruplicates. Statistical significance was assessed by a nonpaired two-tailed Student’s *t* test. **, *P* < 0.01; n.s., not significant. (B) Viable cell numbers in parallel cultures shown in panel A were determined at the indicated time points by trypan blue exclusion. The graph shows mean values and SEM from three replicates collected in one of the experiments shown in panel A. (C) Determination of cellular DNA synthesis in APC- and DMSO-treated cells by EdU click labeling. In parallel to infectivity assays (A), cultures of SupT1-R5 cells were pretreated with APC (1 μM) or DMSO for 20 h. Subsequently, EdU was added for 12 h (corresponding to the 4- to 16-h p.i. interval in infectivity assays). Cells transferred to 8-well chamber dishes were fixed and click labeled. DNA was stained with Hoechst. For each condition, nuclei within 26 randomly selected fields of view were segmented based on the Hoechst signal, and corresponding mean intensities of EdU were determined in Matlab (see Materials and Methods). APC mean, 0.181 × 10^4^, *n* = 817 cells; DMSO mean, 1.647 × 10^4^, *n* = 933 cells. Download FIG S6, TIF file, 0.7 MB. Copyright © 2019 Zila et al. 2019 Zila et al. This content is distributed under the terms of the Creative Commons Attribution 4.0 International license.

10.1128/mBio.02501-19.5 FIG S5 Nuclear entry of CPSF6 binding-defective HIV-1 mutant. The panels show individual fluorescence channels for the enlarged regions 1 to 9 presented in Fig. 6B. Arrowheads indicate CA- and IN.eGFP-positive HIV-1 wild-type (WT) or A77V complexes associated with NPC (filled arrowheads) and IN.eGFP-positive WT complexes inside the nucleus (open arrowheads). Scale bars, 1 μm. Download FIG S5, TIF file, 1.9 MB. Copyright © 2019 Zila et al. 2019 Zila et al. This content is distributed under the terms of the Creative Commons Attribution 4.0 International license.

Next, SupT1-R5 cells were infected for 8 h with wild-type or A77V virions carrying IN.eGFP and then subjected to immunostaining with antiserum against HIV-1 CA and an antibody against NPC (FG repeats). CA-negative complexes were frequently observed inside the nucleus (Fig. 6B, left) and rarely in association with the nuclear envelope in the case of wild-type virus. In contrast, very few HIV-1 subviral complexes were detected in the nuclei of cells infected with the A77V variant (Fig. 6B and C), while a large number of CA-positive subviral complexes accumulated at the nuclear envelope in this case (Fig. 6B and Fig. S5). This phenotype closely resembled that observed in HIV-1 (A77V)-infected MDM (27) and suggested an arrest of subviral complexes directly at the NPC.

**FIG 7:**
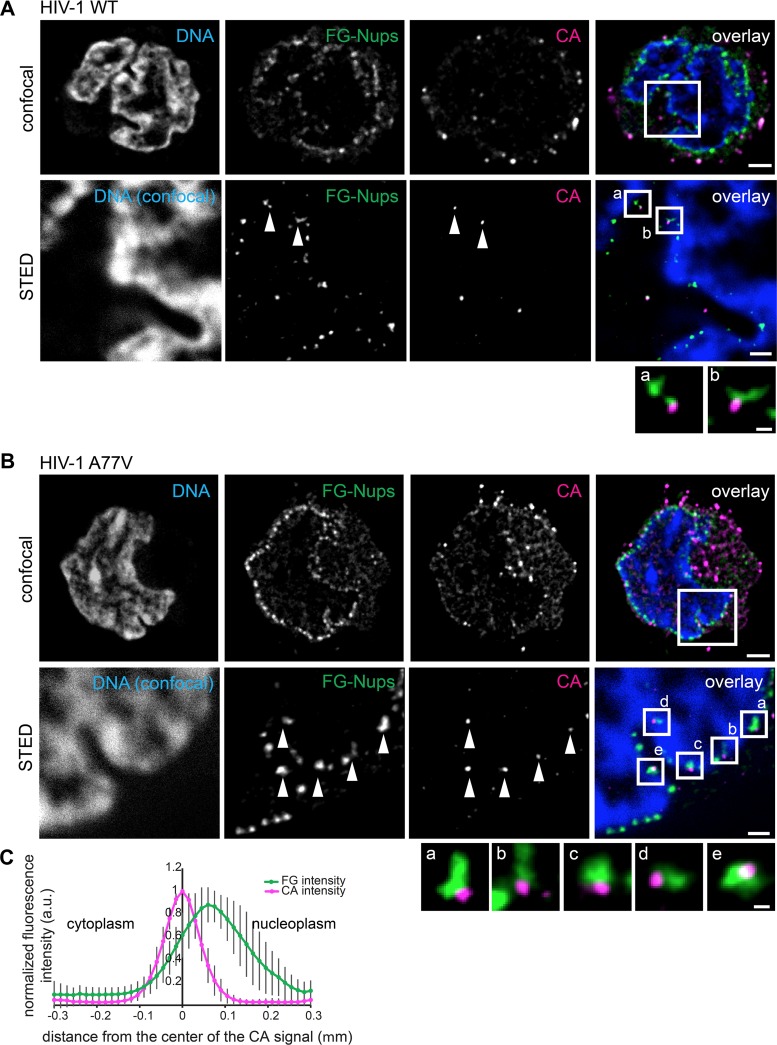
Visualization of HIV-1 wild-type and A77V complexes at the NPC using STED nanoscopy. SupT1-R5 cells were infected with HIV-1_NL4-3_ wild-type (WT) (A) or A77V (B) virions (2 μU of RT/cell) for 8 h at 37°C and immunostained for HIV-1 CA protein (magenta) and NPC (FG repeats, green). DNA was stained with Hoechst (blue). (A and B) Confocal images (upper) and STED images (lower) of the nucleus of a representative infected cell. Arrowheads indicate CA-positive objects colocalizing with NPC proteins. Enlargements of the boxed regions are shown below. Scale bars, 2 μm (confocal images), 500 nm (STED images), and 100 nm (enlarged regions). (C) Averaged line profiles from panel B of selected CA-positive objects (*n* = 30). Error bars represent SD.

To determine the location of subviral complexes at the nuclear envelope with higher precision, we performed dual-color STED nanoscopy (lateral resolution, <50 nm) of SupT1-R5 cells infected with IN.eGFP-labeled wild-type HIV-1 or the A77V variant and immunostained as described above. STED nanoscopy revealed that almost all CA-positive structures at the nuclear envelope were closely associated with NPCs both for wild-type HIV-1 and the A77V variant (Fig. 7A and B). However, many more subviral HIV-1 complexes decorating the nuclear envelope were observed for the A77V variant, always colocalizing with NPCs (Fig. 7B and insets a to e). Line profile analysis of CA- and NPC-specific signal intensities for individual HIV-1 A77V subviral particles revealed a clear overlap of both signals, with CA intensity peaking on the cytoplasmic side of the peak NPC signal at a distance of ∼70 nm (Fig. 7C). Thus, the A77V phenotype in SupT1-R5 cells closely resembled that previously observed in MDM regarding subcellular localization of subviral complexes (27).

## DISCUSSION

Here, we describe a straightforward and robust system for microscopic detection of subviral HIV-1 postfusion complexes using the fluorescent membrane marker mCLING and IN.eGFP-labeled virus, and we apply it to study HIV-1 postentry events in the CD4^+^ T-cell line SupT1-R5. Combination of this approach with specific immunostaining of HIV-1 CA allowed us to determine the relative CA content of subviral complexes in SupT1-R5 cells at different time points p.i. and at different intracellular localizations. mCLING stains only the plasma membrane and plasma membrane-derived vesicles but not other intracellular membranes (51). Furthermore, mCLING staining is stably retained on the membrane after fixation and can be combined with immunofluorescence detection of relevant proteins (51). We validated that mCLING^−^/IN.eGFP^+^ structures truly represent postfusion subviral complexes; appearance of these structures was strongly reduced when fusion was blocked by mutation of the viral fusion peptide or by adding a fusion inhibitor. These mCLING^−^/IN.eGFP^+^ complexes could be easily distinguished from membrane-containing viral particles in the endosomal pathway and were designated cytoplasmic postfusion complexes. mCLING staining moderately enhanced HIV-1 fusion efficiency and infectivity, presumably by a direct effect on the target cell plasma membrane and/or viral envelope. Importantly, however, no cytotoxic effect and no effect on the entry pathway were detected at the mCLING concentration used for our analyses.

We subsequently employed this approach to compare CA signal intensities of HIV-1 subviral postfusion complexes at different subcellular localizations in SupT1-R5 cells with those of complete HIV-1 virions attached to the cell surface in the same cell population. Using a similar approach in HIV-1-infected primary MDM, we had previously shown that CA signal intensities on HIV-1 postfusion complexes are largely similar in the cytoplasm and nucleus, arguing against capsid uncoating in the cytoplasm or at the NPC in this cell type (27). In contrast, loss of a fluorescent CypA derivative, used as an indirect probe for the presence of CA, at the NPC of HIV-1-infected HeLa-derived TZM-bl cells indicated capsid uncoating concomitant with or preceding nuclear entry in this case (19). Here, we showed that cytoplasmic HIV-1 postfusion complexes in SupT1-R5 cells retained ca. 50% of the CA signal intensity of complete virions, independent of their cytoplasmic position. Previous structural and biochemical studies had shown that only ca. 50% of all CA molecules in the virion are needed to form the mature capsid (5–7), and it is currently unclear whether the remaining free CA molecules serve any other function or are only a remnant from the larger amount of Gag molecules needed for immature particle assembly. Our results strongly suggest that these free CA molecules are rapidly lost from the incoming capsid structure, while the CA content of the mature lattice is largely retained throughout cytoplasmic trafficking. Importantly, similar CA signal intensities were detected on cytoplasmic complexes in the cell periphery and on complexes colocalizing with the NPC. Accordingly, the capsid lattice appears to remain largely intact in SupT1-R5 cells until the subviral complex reaches the nuclear envelope. In agreement, several recent live microscopy studies in HeLa cells also did not report significant loss of CA during cytoplasmic passage of subviral HIV-1 complexes that were able to dock at the NPC (19, 58).

Nuclear subviral HIV-1 structures in SupT1-R5 cells appeared to be largely devoid of detectable CA, however, in stark contrast to our previous observations in MDM (21, 26, 27). Side-by-side detection of HIV-1 complexes in SupT1-R5 cells and MDM infected with the same HIV-1 preparation and processed identically and in parallel confirmed this principal difference. Strong CA signals were detected on almost all nuclear HIV-1 complexes in MDM, while corresponding nuclear complexes in SupT1-R5 cells appeared to be devoid of CA. Although immunodetection of nuclear HIV-1 CA is dependent on the experimental condition, this parallel analysis clearly indicates that HIV-1 replication complexes lose all or a large part of their CA content prior to, during, or immediately after nuclear entry in SupT1-R5 cells while travelling largely intact through the cytoplasm until reaching the nuclear pore. Dynamic analysis with highest spatiotemporal resolution (i.e., applying the recently developed Minflux technology [59]) may shed further light on this process.

Comparative analysis of postfusion complexes of wild-type HIV-1 and a CPSF6 binding-defective variant carrying the A77V mutation in CA revealed no obvious difference until subviral complexes reached the nuclear membrane. However, in contrast to wild-type complexes, A77V subviral structures appeared to be largely arrested at the nuclear envelope in direct association with nuclear pores. Very few intranuclear complexes were observed for this variant, and these complexes again appeared CA negative. The large number of nuclear envelope-associated particles was always CA positive, with signal intensity similar to that observed for cytoplasmic postfusion complexes and ca. 50% of that of extracellular bona fide virions. Two-color STED nanoscopy allowed us to determine the exact position of these complexes relative to the FG repeats of nucleoporins. Maximum signal intensity for CA was observed about 70 nm from the maximum of the FG repeat signal toward the cytoplasmic side. Taken together, these results indicate that A77V capsids are similar to the wild type regarding cytoplasmic trafficking and nuclear pore association. They displayed a clear defect in nucleoplasmic entry through intact nuclear pores, however, apparently being arrested at the cytoplasmic side of the NPC. This phenotype is virtually identical to what we previously observed in MDM infected with this variant, where NPC-associated subviral HIV-1 complexes were detected in the same position with respect to the nuclear pore (27). Accordingly, association of the CA lattice with nuclear CPSF6 appears to be important for efficient nucleoplasmic entry through intact nuclear pores not only in MDM but also in this T-cell line.

It appears surprising that the observed strong reduction in the number of nuclear HIV-1 subviral complexes and their accumulation at the nuclear pores is not associated with a stronger infectivity defect in this cell line. The A77V variant showed only mildly reduced specific infectivity in SupT1-R5 cells, indicating that the CA-CPSF6 association is dispensable for infection and, thus, also for access to the host genome in these cells. A moderate reduction of infectivity had also been observed for the A77V variant in MDM, and again this did not seem to reflect the strong phenotype observed for nuclear entry (27). Based on these results, we speculated that CPSF6 binding-defective HIV-1 complexes are still capable of reaching the nuclear basket in MDM and integrating into NPC-proximal chromatin without reaching further into the nucleoplasm (27). This hypothesis is consistent with another recent study suggesting a nucleoplasm trafficking defect for a CPSF6 binding-defective variant (36).

In contrast to primary MDM, SupT1-R5 cells are proliferating cells, and residual infectivity of the A77V variant also could be due to nuclear envelope breakdown upon mitosis. However, specific infectivity was equally affected for wild-type HIV-1 and the A77V variant in APC-arrested cells, indicating that subviral complexes trapped at the NPC can access chromatin without nuclear envelope breakdown, as had been shown for MDM. This phenotype is consistent with previous reports showing that the CPSF6 binding-defective CA variant N74D of HIV-1 retains infectivity in APC-treated HeLa cells (60).

Further analysis regarding structural details of the NPC-arrested and nuclear subviral HIV-1 complexes will require higher-resolution ultrastructural methods. Obviously, CA signal intensity only provides information on the CA content of the detected structure, not on the overall structural integrity of the capsid lattice. The experimental system described in the current report appears ideally suited for such studies, since it allows extension toward correlative light and electron microscopy to detect and structurally analyze HIV-1 postfusion complexes at different subcellular localizations.

## MATERIALS AND METHODS

### Cell cultures.

The human T lymphoblast cell line SupT1-R5 (stably expressing exogenous CCR5 under puromycin selection; a kind gift from R. Doms, University of PA, USA; certified by Eurofins according to DAkkS ISO 9001:2008) was cultivated at 37°C in a humidified incubator with a 5% CO_2_ atmosphere, using RPMI 1640 medium with GlutaMAX (ThermoFisher) supplemented with 10% fetal bovine serum (FBS; Merck), 50 U/ml of penicillin, 50 μg/ml of streptomycin (ThermoFisher), and 0.3 μg/ml puromycin (Merck). Human embryonic kidney 293T cells (HEK 293T) were maintained in Dulbecco’s modified Eagle medium (DMEM; ThermoFisher) supplemented with 10% FBS, 50 U/ml penicillin, and 50 μg/ml streptomycin. For preparation of monocyte-derived macrophages (MDM), human peripheral blood mononuclear cells (PBMC) were isolated from buffy coats as described in Bejarano et al. (26). Buffy coats were obtained from healthy anonymous blood donors at the Heidelberg University Hospital Blood Bank according to the regulations of the local ethics committee. Adhered monocytes were subsequently cultured and differentiated into MDM in RPMI 1640 medium supplemented with 10% heat-inactivated FBS, antibiotics, and 5% human AB serum (ThermoFisher) for 7 to 10 days; medium was replaced every three days.

### Plasmids.

Plasmid pCHIV for production of replication-incompetent HIV-1 particles (48), the proviral plasmids pNLC4-3 (61), pNLC4-3ΔEnv (40), pNL4-3ΔEnv (27), and pNL4-3ΔEnv-A77V (27), the Env expression vector pCAGGS.NL4-3-Xba (62), and plasmid pEnv(Fus)V2E for production of fusion-incompetent Env (40) were described previously. Plasmid pEnv-4059 (54), encoding an R5-tropic Env from a clinical HIV-1 isolate, was kindly provided by R. Swanstrom (University of North Carolina, Chapel Hill, NC, USA). Plasmid pVpr.IN.eGFP (53), encoding a Vpr.IN.eGFP fusion protein with an HIV-1 protease recognition site between Vpr and IN, was kindly provided by A. Cereseto (CIBIO, Mattareo, Italy). Plasmid pMM310 (63), encoding a β-lactamase-Vpr fusion protein, was kindly provided by N. R. Landau (New York University, NY, USA).

### Antibodies and reagents.

Rabbit polyclonal antiserum against HIV-1 CA was raised against purified recombinant protein (in house) and was used at a dilution of 1:1,000. Mouse monoclonal antibody against NPC proteins (ab24609; Abcam) was used at a 1:200 dilution. Affinity-purified goat antibody against Nup358/Ran-BP2 (64), kindly provided by F. Melchior (ZMBH, Heidelberg University, Germany), was used at a dilution of 1:200. Mouse monoclonal antibody against lamin A/C (sc-7292; Santa Cruz) was used at a dilution of 1:100. For detection of CA by flow cytometry, fluorescein isothiocyanate (FITC)-conjugated mouse monoclonal antibody KC57 (Beckman Coulter) was used at a dilution of 1:100. The following secondary antibodies were used: goat anti-rabbit IgG, donkey anti-rabbit IgG, and donkey anti-mouse IgG, conjugated with Alexa Fluor 488, 568, and 647, respectively (all purchased from ThermoFisher), at a 1:1,000 dilution. For STED microscopy, goat anti-rabbit IgG conjugated with Atto594 and goat anti-mouse conjugated with STAR RED (both purchased from Abberior GmbH, Germany) were used at a 1:200 dilution. DNA was stained with Hoechst (ThermoFisher).

A stock solution of 50 μM mCLING labeled with Atto647N (710006AT1; Synaptic Systems, Göttingen, Germany) in phosphate-buffered saline (PBS) was stored at –80°C. A stock solution of 10 mM efavirenz (obtained through the AIDS Research and Reference Reagent Program, Division AIDS, NIAID) or PF74 (ThermoFisher) was prepared in dimethyl sulfoxide and stored at –20°C. A 10 mM stock solution of raltegravir (obtained through the AIDS Research and Reference Reagent Program, Division AIDS, NIAID) or T-20 (enfuvirtide; Roche) was prepared in H_2_O and stored at –20°C. A stock solution of 6 mM aphidicolin (APC) (Merck) was prepared in dimethyl sulfoxide (DMSO) and stored at –20°C.

### Virus and virus-like particle production.

For production of HIV-1 virions and virus-like particles, HEK 293T cells grown on 175-cm^2^ side-bottom tissue culture flasks were transfected using calcium phosphate precipitation according to standard procedures. For production of infectious HIV-1, cells were transfected with 70 μg pNLC4-3 per 175-cm^2^ flask. To produce infectious (HIV-1_NL4-3_) or non-replication-competent (HIV-1_CHIV_) particles labeled with IN.eGFP, cells were cotransfected with 61 μg of pNLC4-3 or pCHIV and 9 μg of pVpr.IN.eGFP per 175-cm^2^ flask. To produce Vpr.β-lactamase-carrying HIV-1_NL4-3_, cells were cotransfected with 61 μg of pNLC4-3 and 9 μg of pMM310 per flask. For production of pseudotyped HIV-1, cells were cotransfected with 61 μg of pNLC4-3ΔEnv and 9 μg of the Env expression vector pCAGGS.NL4-3-Xba or pEnv(Fus)V2E. To produce pseudotyped R5-tropic HIV-1 viral particles, cells were cotransfected with 61 μg of pNL4-3ΔEnv or pNL4-3ΔEnv-A77V and 9 μg of pEnv-4059; to produce their IN.eGFP-labeled variants, cell were cotransfected with 56 μg of pNL4-3ΔEnv or pNL4-3ΔEnv-A77V, 7 μg of pEnv-4059, and 7 μg of pVpr.IN.eGFP per flask. Culture media from virus-producing cells were harvested at 44 to 48 h posttransfection and cleared by filtration through a 0.45-μm nitrocellulose filter, and particles from media were concentrated by ultracentrifugation through a 20% (wt/wt) sucrose cushion at 125,000 × *g* for 90 min at 4°C. For ddPCR analysis, virus-containing medium from producing cells was treated with 15 U/ml DNase I (Merck) and 10 mM MgCl_2_ for 5 h at 37°C prior to ultracentrifugation. Particles were resuspended in PBS containing 10% FBS and 10 mM HEPES (pH 7.2) and stored in aliquots at −80°C. Particle-associated RT activity was determined by SG-PERT (SYBR green-based product-enhanced reverse transcription assay) (65).

### Infectivity assays.

SupT1-R5 cells were distributed into U-bottom 96-well plates (3 × 10^5^ cells/well; 650180; Greiner Bio-One) and incubated with the indicated amounts of the respective virus derivative for 90 min at 16°C for virus adsorption. Subsequently, the inoculum was removed, fresh medium was added, and the temperature was shifted to 37°C to initiate virus entry. Cells were incubated at 37°C for the indicated time period. To monitor the kinetics of HIV-1 productive entry in SupT1-R5 cells, T-20 (50 μM final concentration), efavirenz (5 μM), PF74 (2 μM), or raltegravir (5 μM) was added to HIV-1_NL4-3_-infected cells (2.4 μU of RT/cell) for the indicated time periods. Noninfected cells, cells infected with equal amounts of HIV-1_NL4-3_ΔEnv particles, and untreated cells were used as controls. To monitor the effect of mCLING labeling on HIV-1 productive entry, cells were incubated with HIV-1_NL4-3_ (2.8 μU of RT/cell) or equal amounts of HIV-1_NL4-3_ΔEnv and incubated at 16°C as described above. After adsorption, inoculum was changed for fresh medium or medium containing the indicated concentration of mCLING.Atto647N (Synaptic Systems), and incubation at 16°C was prolonged for an additional 10 min to achieve plasma membrane labeling. The temperature was then shifted to 37°C, and cells were incubated for 2 h before addition of 50 μM T-20. To examine the contribution of endocytosis to HIV-1 infection in the presence of mCLING, cells were preincubated in medium containing mCLING.Atto647N (2 μM) for 10 min at 22°C and then incubated with HIV-1_NL4-3_ (6.9 μU of RT/cell) for 4 h at 22°C or 37°C. After that, T-20 (50 μM) was added, and cells were incubated for an additional hour at 22°C and subsequently shifted to 37°C. Noninfected and unstained cells were used as controls. To determine the importance of CPSF6 binding for virus replication, SupT1-R5 cells were infected with two different concentrations of HIV-1_NL4-3_ CA wild-type or A77V virions (2 or 4 μU of RT/cell) and incubated for 5 h at 37°C before addition of T-20 (50 μM). To examine the effect of cell cycle arrest on HIV-1 infectivity, cells were pretreated with APC (1 μM) for 16 h at 37°C and infected with wild-type or A77V virions (both at 1 μU of RT/cell). Cells pretreated and infected in the presence of DMSO and mock-infected cells were used as controls. Inoculum was exchanged for fresh medium supplemented with T-20 (50 μM) and APC (1 μM) at 24 h p.i., and infectivity was scored by flow cytometry at 48 h p.i. For this, cells were fixed with 4% formaldehyde (FA) in PBS, pH 7.4, for 90 min at room temperature and immunostained for intracellular HIV-1 CA for 30 min at 4°C using KC57-FITC antibody (Beckman Coulter) diluted in 0.1% Triton X-100, 0.1 mg/ml bovine serum albumin in PBS. Cells were analyzed by flow cytometry using a BD FACSVerse flow cytometer (BD Biosciences).

### Fusion assays.

Efficiency of HIV-1 cytosolic entry was measured using a previously described β-lactamase HIV-1 fusion assay (52). SupT1-R5 cells were distributed into 96-well plates (3 × 10^5^ cells/well; U-bottom; Greiner Bio-One) and incubated with Vpr.β-lactamase-carrying HIV-1_NL4-3_ virions (1.1 μU of RT/cell) for 90 min at 16°C for adsorption to the cell surface. Subsequently, inoculum was replaced by fresh culture medium (control) or medium containing the indicated concentration of mCLING.Atto647N, and incubation at 16°C was prolonged for an additional 10 min for plasma membrane staining. Temperature was shifted to 37°C for 2 h, and then cells were washed with PBS and incubated in the dark at room temperature in CO_2_-independent medium (ThermoFisher) supplemented with 2 μM CCF2-AM substrate (prepared according to the manufacturer’s instructions; ThermoFisher) and 2.5 mM probenecid (ThermoFisher) for 16 h. Cells were then fixed with 4% FA in PBS for 60 min at room temperature and analyzed by flow cytometry using a BD FACSVerse flow cytometer (BD Biosciences). CCF2 fluorescence was excited at 405 nm, and emission maxima at 447 nm (CCF2blue) and 520 nm (CCF2green) were recorded using appropriate filter settings.

### Cell viability and proliferation assays.

To test the effect of mCLING staining on cell viability, SupT1-R5 cells were distributed into 96-well plates (3 × 10^5^ cells/well; U-bottom; Greiner Bio-One) and incubated in medium supplemented with the indicated concentration of mCLING.Atto647N for 3 h at 37°C. The cell suspension was analyzed directly after staining or following incubation for an additional 48 h at 37°C in the absence of the fluorescent probe. To test the effect of APC treatment on cell proliferation, SupT1-R5 cells were stained with Trypan blue using standard procedures and counted at different time points after APC addition. To determine APC effects on cellular DNA synthesis, SupT1-R5 cells were arrested with 1 μM APC (or DMSO as a control) for 20 h, after which 10 μM EdU was added for 12 h. Cells transferred to 8-well chamber dishes were fixed and click labeled using the Click-iT EdU-Alexa Fluor 647 imaging kit (ThermoFisher) according to the manufacturer’s instructions. Nuclei were segmented based on the Hoechst signal using Ilastik (66) and watershed separated using Fiji (67), and corresponding mean intensities of EdU were determined in Matlab (MathWorks, MA, USA) and plotted using the visualization toolbox Gramm (68).

### Detection of HIV-1 RT products by ddPCR.

SupT1-R5 cells in 96-well plates (3 × 10^5^ cells/well; U-bottom; Greiner Bio-One) were infected with HIV-1_NL4-3_ or HIV-1_CHIV_ (both at 5.6 μU of RT/cell) and incubated at 37°C. At selected times postinfection, cells were washed with PBS and lysed overnight at 55°C using an in-house lysis buffer (10 mM Tris-HCl, pH 9.0, 0.1% Triton X-100, 400 μg/ml proteinase K [ThermoFisher]). Proteinase K was inactivated by incubation at 95°C for 10 min, and lysates were stored at –20°C or diluted and used for RT product detection by digital droplet PCR (ddPCR) (47, 69). Late RT products were detected with a set of primers/probe annealing to the *gag* open reading frame (forward, 5′-CATGTTTTCAGCATTATCAGAAGGA-3′; reverse, 5′-TGCTTGATGTCCCCCCACT-3′; probe, 5′-6-carboxyfluorescein [FAM]-CCACCCCACAAGATTTAAACACCATGCTAA-black hole quencher 1 [BHQ1]-3′ [70]). Another set of primers/probe was used to detect 2-LTR circles (forward, 5′-CTAACTAGGGAACCCACTGCT-3′; reverse, 5′-GTAGTTCTGCCAATCAGGGAA-3′; probe, 5′-FAM-AGCCTCAATAAAGCTTGCCTTGAGTGC-BHQ1-3′ [71]). To normalize for sample input, the single-copy host gene encoding RNase P protein subunit p30 (RPP30) was quantified (forward, 5′-GATTTGGACCTGCGAGCG-3′; reverse, 5′-GCGGCTGTCTCCACAAGT-3′; probe, 5′-FAM-CTGACCTGAAGGCTCT-BHQ1-3′ [69]).

For each sample, 20-μl reaction mixtures containing 2 μl of lysate (*gag* and RPP30, 1:5 prediluted, 2-LTR undiluted), 900 nM primer, 200 nM probe, 1× ddPCR Supermix for probes (no dUTP) (Bio-Rad) for *gag* and RPP30 amplification and ddPCR Supermix for residual DNA quantification (Bio-Rad) for 2-LTR amplification were prepared, and droplets were generated using the QX200 droplet generator and droplet generation oil for probes (Bio-Rad). Subsequently, droplets were transferred to a 96-well microplate for PCR amplification. PCR was performed starting with initial denaturation and stabilization at 95°C for 10 min, followed by 40 cycles of denaturation at 94°C for 30 s, annealing/extension at 57°C for 60 s, and finishing with 10 min at 98°C. Stabilized droplets were analyzed in a QX200 droplet reader (Bio-Rad) using the settings for absolute quantification (*gag* and RPP30) or residual DNA quantification (2-LTR). Results were analyzed using QuantaSoft software (Bio-Rad), and copy numbers of HIV-1-derived DNA were normalized to the copy numbers of the RPP30 housekeeping gene.

### Immunofluorescence staining.

For confocal or superresolution STED microscopy, SupT1-R5 cells were distributed into 96-well plates (3 × 10^5^ cells/well; U-bottom; Greiner Bio-One) and incubated with IN.eGFP-carrying HIV-1_NL4-3_ or HIV-1_CHIV_ for 90 min at 16°C. For detection of postfusion complexes, mCLING.Atto647N was added after adsorption at a final concentration of 2 μM, and cells were incubated for an additional 10 min at 16°C. Cells were then transferred to polyethylenimine (PEI)-coated wells of glass-bottom 8-well chamber slides (155411 [LabTek; ThermoFisher] or 80827 [Ibidi]), and temperature was shifted to 37°C to initiate virus entry. Alternatively, cells were seeded in PEI-coated 8-well chamber slides and virus adsorption was performed directly, followed by mCLING staining at 16°C. At the indicated time points, cells were fixed for 15 min at room temperature either with 4% FA in PBS or (for mCLING-labeled samples) with 4% FA and 0.2% glutaraldehyde in PBS to ensure retention of mCLING membrane localization in fixed and permeabilized samples (51). For immunodetection, cells were permeabilized with 0.5% Triton X-100 (5 to 10 min) in PBS and then washed with PBS and blocked for 30 min with 2% BSA in PBS. Immunostaining with primary and secondary antibody was carried out for 1 h each. DNA was stained with 5 μg/ml Hoechst in PBS for 30 min.

To detect CA as previously reported for MDM (26, 27), MDM (5 × 10^4^) or SupT1-R5 (3 × 10^5^) cells in wells of 8-well chamber slides (80827; Ibidi) were infected with IN.eGFP-carrying HIV-1_NL4-3_ (6.7 × 10^5^ μU of RT/well) pseudotyped with an R5-tropic envelope for 5.5 h or 48 h at 37°C, respectively. Samples were fixed with 4% FA in PBS (15 min), permeabilized with 0.5% Triton X-100 in PBS for 20 min, and treated for 30 min with the reaction cocktail from the Click-iT EdU-Alexa Fluor 647 imaging kit (ThermoFisher), prepared according to the manufacturer’s instructions. Cells were then washed with PBS and processed for immunostaining as described above.

### Microscopy and image processing.

Multichannel z-series of infected cells were acquired by a Nikon Ti PerkinElmer UltraVIEW VoX 3D spinning-disc confocal microscope (Perkin Elmer, Waltham, MA) using a 100× oil immersion objective (numeric aperture [NA], 1.49; Nikon), with a z-spacing of 200 nm and excitation with the 405-, 488-, 561-, and 633-nm laser lines. Camera background subtraction, illumination correction, gauss filtering, and cropping were performed using Fiji software (67). Where indicated, acquired images were subsequently deconvolved by Autoquant X3 (Media Cybernetics, Rockville, MD) using the theoretical point spread function and the constrained maximal likelihood estimation (CMLE) algorithm with 10 iterations and a signal-to-noise ratio of 20. To quantify virus binding, particles associated with the cell surface were determined from the maximum-intensity projections of z-stacks using spot detector of the software Icy (72).

To validate mCLING-based detection of HIV-1 postfusion complexes, 8-μm-thick z-stacks through the volume of cells were acquired with a Leica TCS SP8 confocal laser scanning microscope (Leica Microsystems, Germany) using a 63× oil immersion objective (NA, 1.49; Leica, Wetzlar, Germany), with a z-spacing of 500 nm and excitation with the 488-, 561-, and 633-nm laser lines. z-stacks were acquired in 9 tiled positions (3 by 3), which were then stitched together (representing an area of 0.5 mm^2^).

Single- or dual-color stimulated emission depletion (STED) microscopy was performed with a 775 STED system (Abberior Instruments GmbH, Göttingen, Germany), using a 100× oil immersion objective (NA, 1.4; Olympus UPlanSApo) and excitation with the 590- and 640-nm laser lines. Nominal STED laser power was set to 10% of the maximal power of 3 mW with pixel dwell time of 20 to 30 μs and 15-nm pixel size. Dual-color STED images were linearly deconvolved with a Lorentzian function (full width at half maximum, 50 nm) using the software Imspector (Abberior Instruments GmbH).

### Image analysis.

To measure and quantify the fluorescent signal intensities from cell-associated HIV-1 structures, a 3D volume of infected cells was reconstructed from acquired z-stacks using Imaris software (Bitplane AG, Zürich, Switzerland). Individual HIV-1 CA and/or IN.eGFP signals were automatically detected using the spot detector Imaris module, creating for each distinct fluorescent signal a 3D ellipsoid object with a 300-nm estimated diameter in *x-y* dimensions and 600 nm estimated diameter in *z* dimension. The median signal intensity within objects was quantitated for the mCLING or HIV-1 CA channel. To validate mCLING-based detection of HIV-1 postfusion complexes, the objects containing IN.eGFP signal and having median mCLING intensity lower than 1 to 2 photons were considered to be true postfusion complexes.
