# Supplementary figures and images for: Analysis of CA Content and CPSF6 Dependence of Early HIV-1 Replication Complexes in SupT1-R5 Cells
Source: mBio. 2019 Nov 5;10(6):e02501-19. doi: 10.1128/mBio.02501-19 (PMC6831778; doi:10.1128/mBio.02501-19)

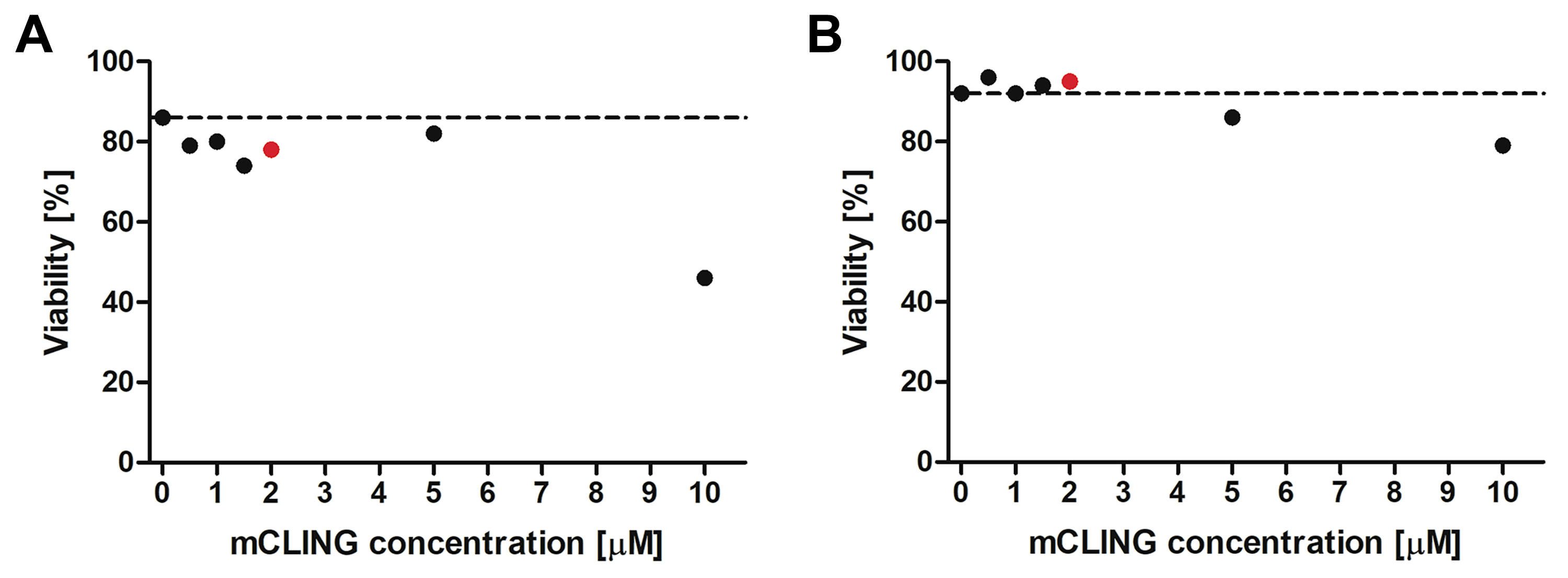

Supplement: FIG S1 [file mBio.02501-19-sf001.tif]

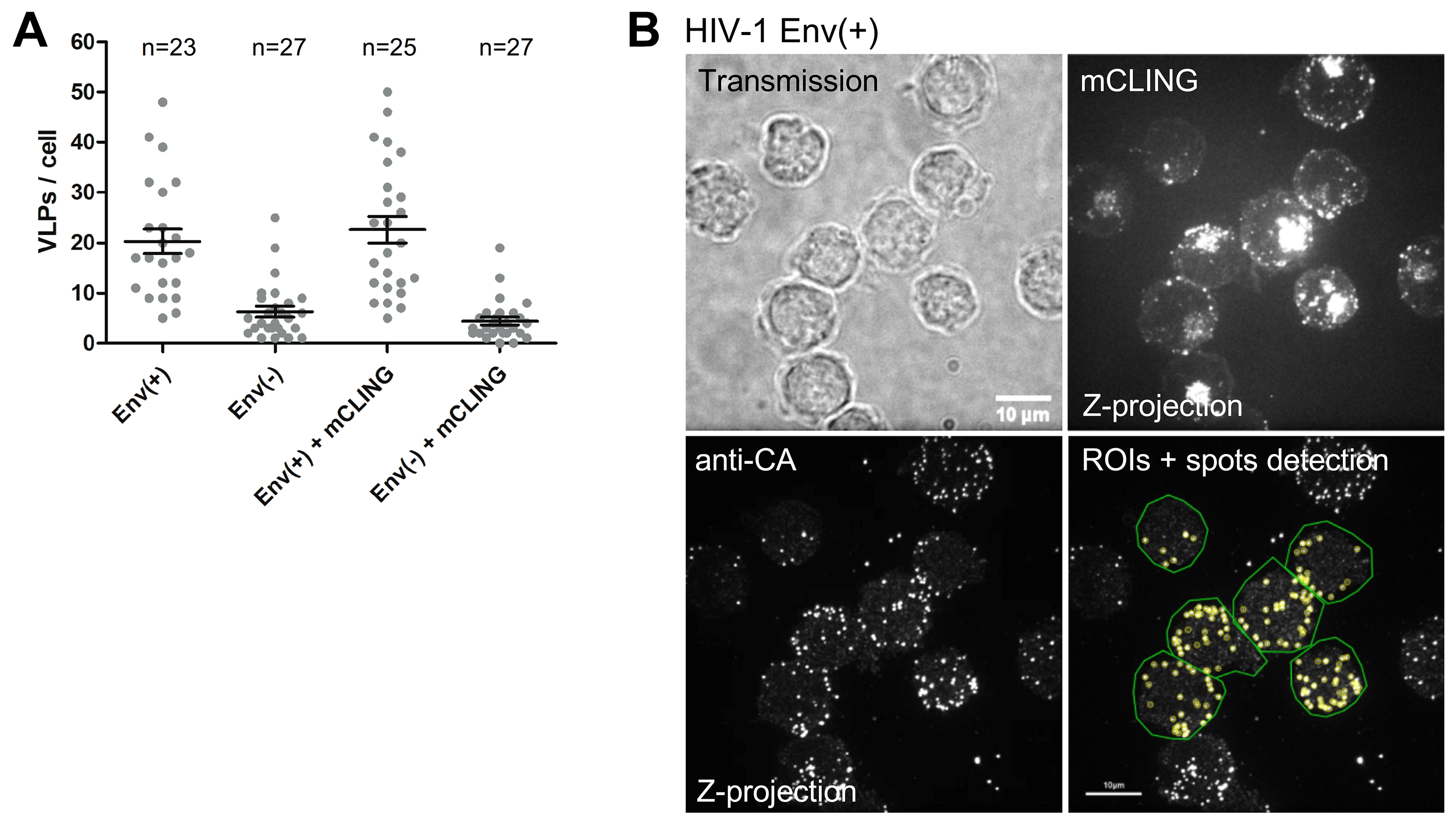

Supplement: FIG S2 [file mBio.02501-19-sf002.tif]

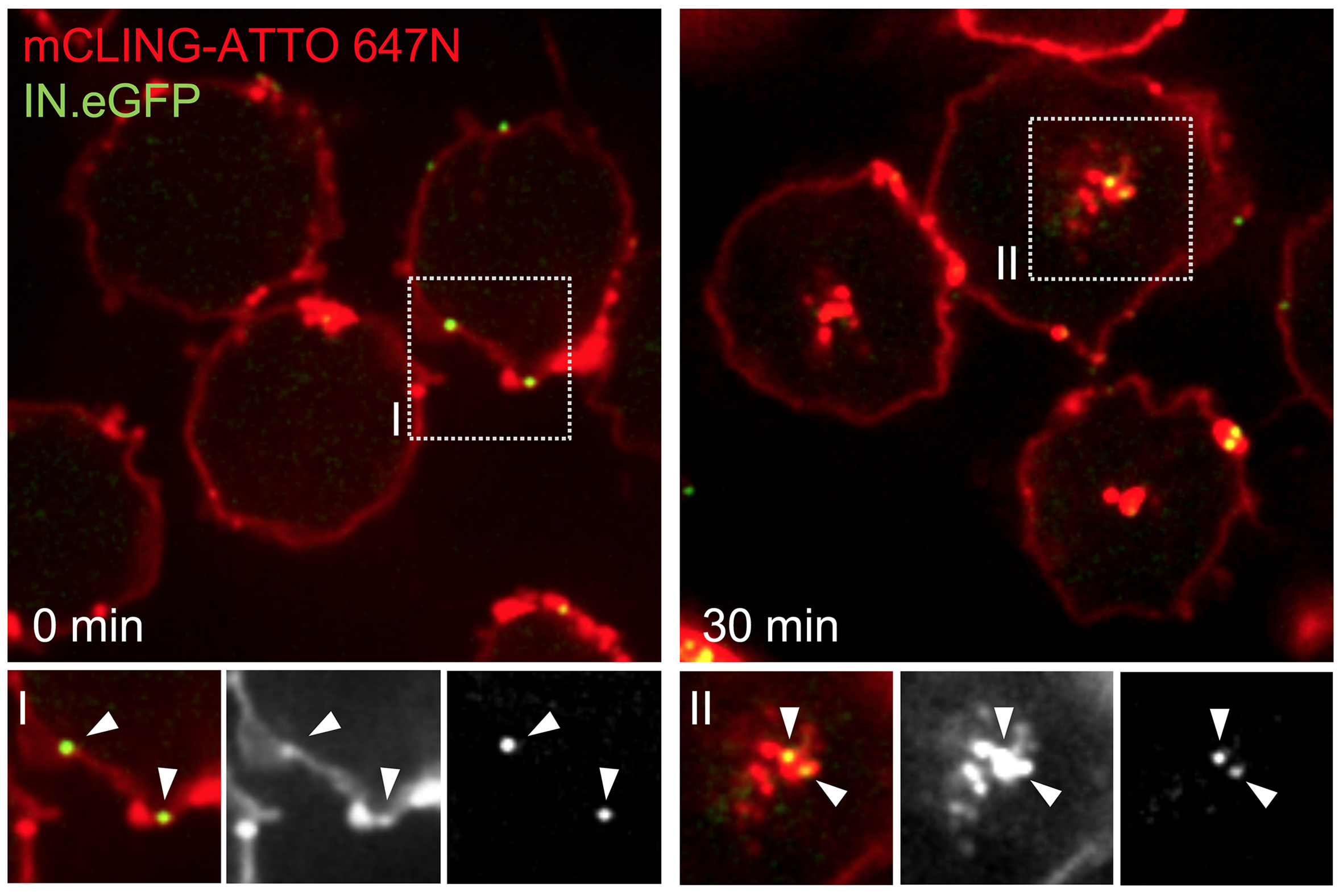

Supplement: FIG S3 [file mBio.02501-19-sf003.tif]

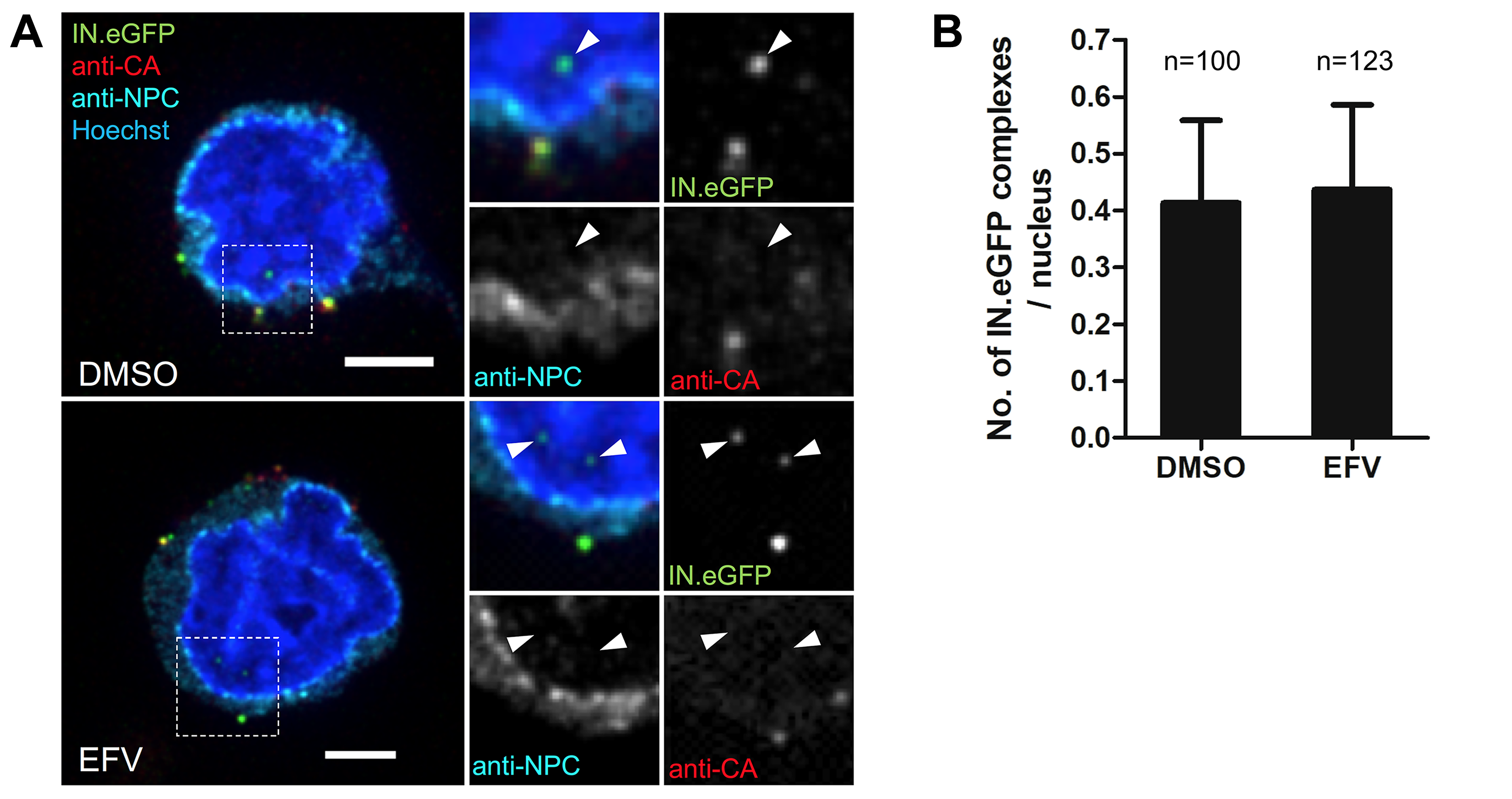

Supplement: FIG S4 [file mBio.02501-19-sf004.tif]

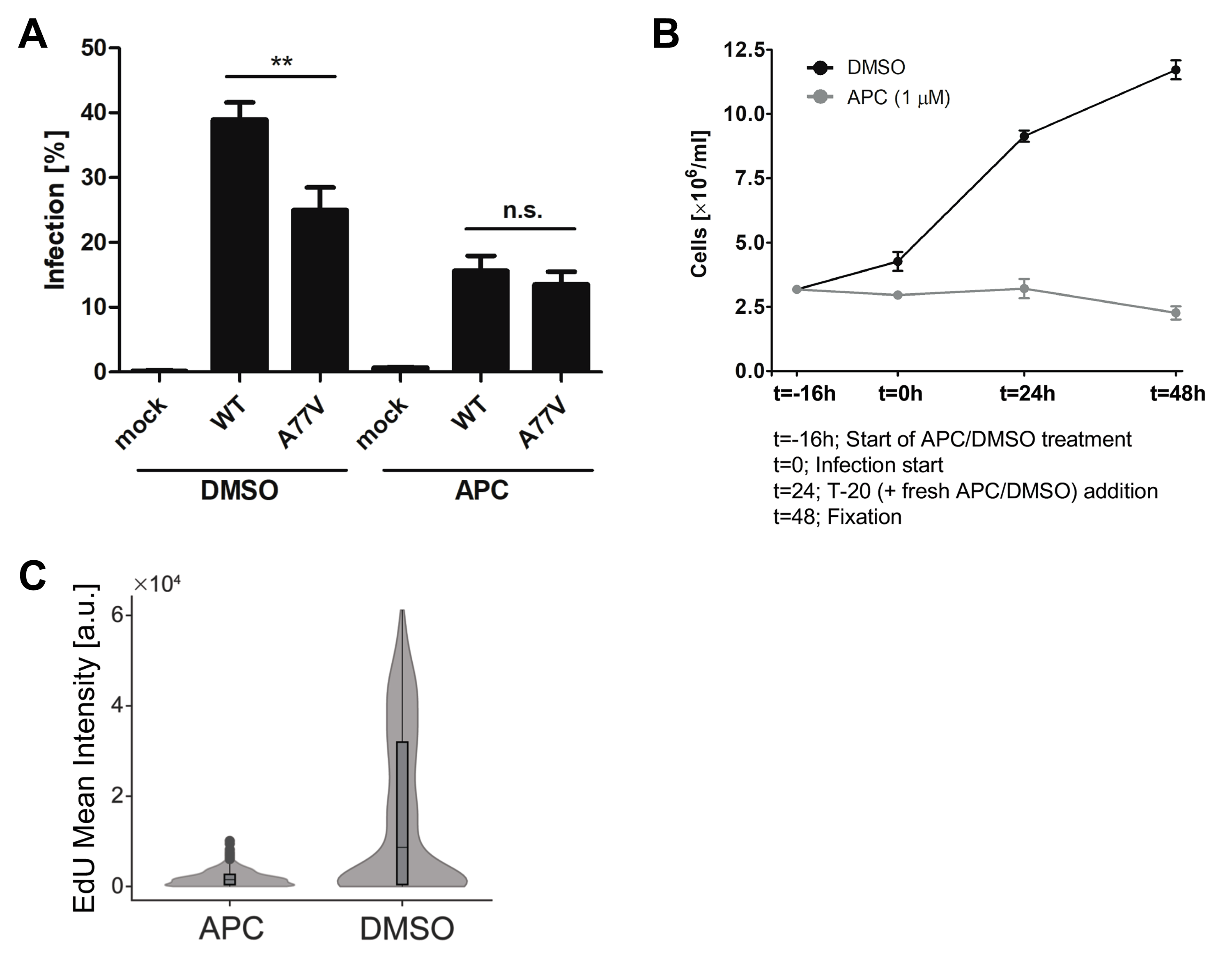

Supplement: FIG S6 [file mBio.02501-19-sf006.tif]

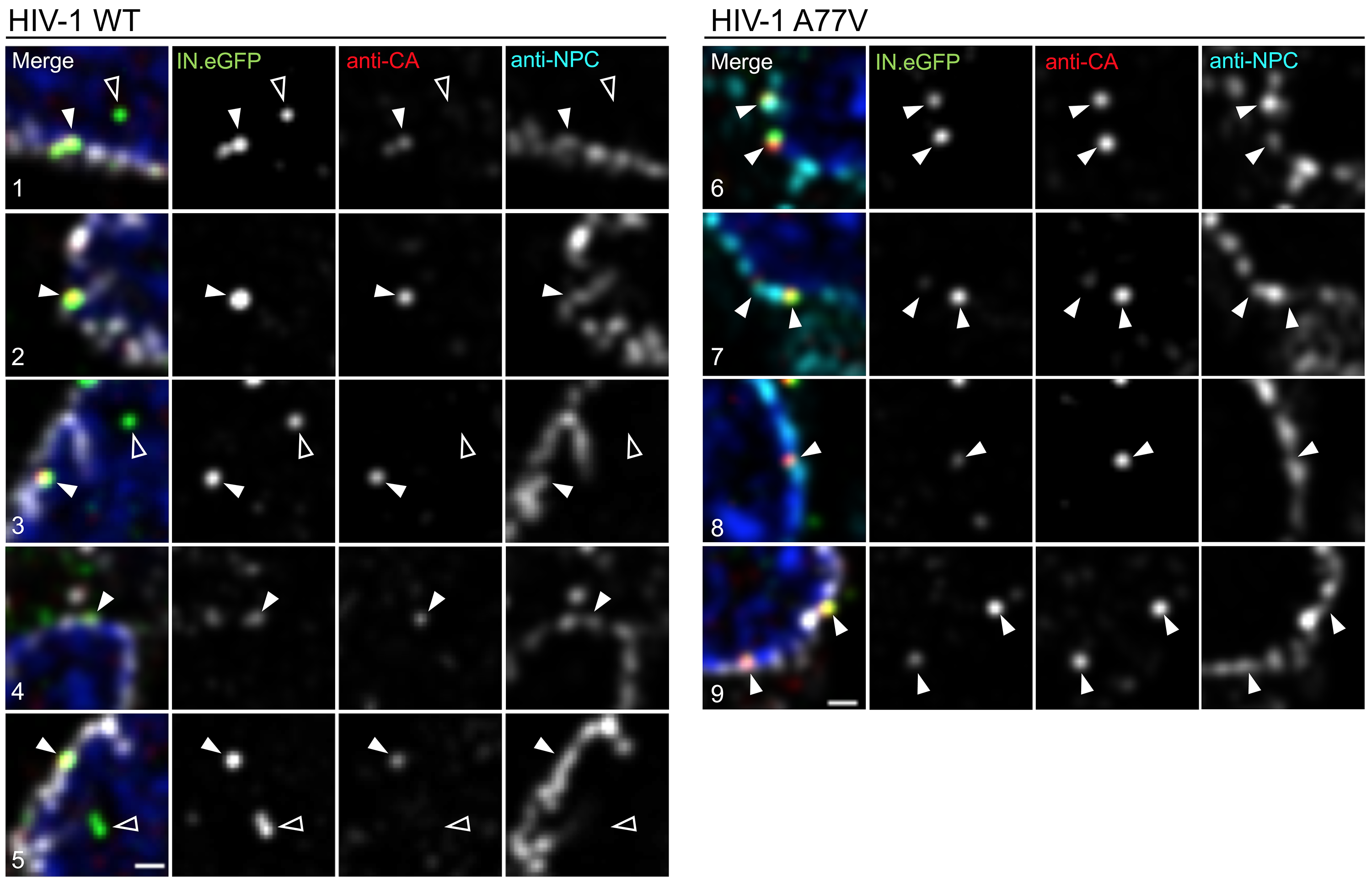

Supplement: FIG S5 [file mBio.02501-19-sf005.tif]
